# Model experiments and theory of flame front propagation in forest fire

**DOI:** 10.1038/s41598-026-47836-5

**Published:** 2026-04-21

**Authors:** Alexander L. Yarin, Wenshuo Zhang, Eyal Zussman

**Affiliations:** 1 Department of Mechanical and Industrial Engineering, University of Illinois at Chicago, 842 W. Taylor St, Chicago, IL 60607-7022 USA; 2 Faculty of Mechanical Engineering, The Technion- Israel Institute of Technology, Haifa, 32000 Israel

**Keywords:** Fire, Flame Propagation, Forest, Model Experiments, Modeling, Ecology, Ecology, Engineering, Environmental sciences, Natural hazards

## Abstract

To quantify how inter-source spacing (“spatial step”), inclination angle (slope), and wind affect flame-propagation velocity, model experiments were conducted in the present work. Propagation velocities and flame morphologies were quantified and visualized. The measured dependence of velocity on wind speed, slope, and source spacing is then used to formulate and illustrate a theory for forest-fire flame-front propagation affected by wind over flat and hilly terrains. The results demonstrate dramatic effect of wind on flame propagation and the overall configuration of the flame front, as well as a significant difference between highly volatile and flammable versus less flammable fuels. The model with continuous flame front developed in the present work approximates the cases where the tree-to-tree distances and the characteristic flame transfer times between trees are much smaller and shorter, respectively, than the sizes and evolution times of forest fire. The model implies that the flame propagation is sustained by volatiles rapidly released by plants in fire, which sustain fire propagation serving as a fuel mixing in a thin flame front with oxidizer from the ambient air like in diffusion flames. It is also implied that the flame front can be considered as continuous on its scale which is much larger than the tree-to-tree distances (that means the so-called diffusion approximation in the mathematical sense). The theoretical predictions reveal that the flame front configuration is strongly affected by landscape topography and wind, much more than by the initial ignition configuration. It should be emphasized that (i) the model lab-scale experiments here only elucidate on the physical level the main effects which are to be expected from the large-scale experiments: that flame propagation velocity strongly depends on fuel volatility, spacing between sources, terrain inclination, and wind presence. (ii) The large-scale predictive capability of the quasi-physical theoretical/numerical model developed here is still to be verified by fitting the two lumped parameters of the model to large-scale data. The latter is currently hardly possible because the available published data on distillation stage of forest fires are scarce and typically lacking many important details in their totality, like slopes and wind speed, the type of wood involved, the volatile and water content in the wood, etc. (iii) Accordingly, the present numerical results, albeit some of them were obtained for a real kilometer-scale landscape, are illustrative in nature. Still, in future widening of a detailed experimental data bank and its interpretation by such methods as Machine Learning and AI will allow a reliable and detailed verification of the present quasi-physical theoretical/numerical approach. That will yield potentially impactful insights into occurrence of forest fires around urban areas as well as of urban fires, with the present quasi-physical model being used for monitoring, advanced planning and mitigation.

## Introduction

 Forests cover about 30% of the world land area, contribute significantly to its ecosystem, reducing carbon dioxide emissions and strengthening soils, as well as providing wildlife and humans with a variety of living resources. However, forests are threatened by both deliberate deforestation (caused by different reasons such as unsustainable logging and ranching) and uncontrolled wildfires^1–3^. Recent years have revealed a clear evolution toward more erratic, large-scale, and high-power fire behavior, often referred to as extreme or mega-fires^4^. Usually there are four main reasons that can cause a forest fire: lightning, sparks, vegetation spontaneous combustion, and volcanic eruptions^5^.

Different types of fires encompass fires over grassland/spinifex or pine needle fuel beds^6,7^, or forest fires^8^. The latter are typically sub-divided into five main stages, such as ignition, flaming, glowing, smoldering and extinction, and some sub-classes of these stages. The present work has in focus the flaming, drying/distilling stage of forest fires (crown-fire or canopy-level propagation), when volatiles (fuel vapor) and water vapor are ejected from wood directly into the surrounding flame providing heat for such eruptions^9^.

Forest fires are inherently uncontrollable and propagate freely, making it crucial to comprehensively understand the mechanisms of flame propagation within forest ecosystems, whether in canopy or via trunks. This includes examining the factors influencing propagation and their interrelationships. In the 20th century, several physical models were found to describe the mechanism of flame propagation from several aspects, such as radiative transfer^10–14^, heat transfer^15,16^, and energy flux conservation^17^. These theoretical models, which depict flame transport, require a number of input parameters. In particular, empirical correlations are used for estimating flame geometry and using the latter, heat transfer estimates are used to predict the flame spread rate^13,18–20^. However, some key input parameters, such as flame height, are hardly measured under natural conditions and cannot always remain constant. Also, the composition and properties of flammable materials exhibit variability across different locations and seasons. Consequently, flame propagation models are unable to address all potential issues, even though some of them involve massive numerical simulations, and their limitations remain evident.

Experimental studies on fire propagation, which complement theoretical research, are conducted either in laboratory settings or under natural conditions^21–24^. Pine needles^25,26^ and oak leaves^27^, which are prevalent in forest ecosystems, were utilized in combustion experiments to simulate fire behavior. The experimental data derived from these trials do not always fully capture flame propagation in natural forest settings, as the spatial distribution of trees, which is important for crown fire propagation, remains an unaccounted-for variable. It should be emphasized that for surface fires, fuel present on the ground, e.g., pine needles, twigs, oak leaves, etc. is very important. Additional natural conditions, such as slope and wind effects, were also incorporated into the experimental design, with particular emphasis on their individual and combined impacts^28–30^.

Forest fire propagation prediction has been a focus of numerous research groups, employing diverse methodologies. The most fundamental approach relies on partial differential equations governing mass, momentum, heat, and species balances in turbulent reacting flows, along with buoyancy and radiation effects^31–34^. Similar efforts have addressed the impact of global explosions on rapid forest destruction and burning during major incidents^35^. However, this fundamental approach demands extensive numerical simulations and faces challenges due to the variability of fuel types, moisture levels, and the need for significant empirical data, which is often insufficient for accurate turbulent flow modeling under the conditions of forest fire. Consequently, operational models have been widely adopted, utilizing empirical equations that incorporate fuel types, moisture levels^36^, landscape slopes, and wind conditions to predict flame front propagation^37–46^. Note also an empirical so-called ‘B-number model’ utilizing some elements from the theory of flow near stagnation point and applying them for predicting the ignition time and flame over linear, discrete fuel arrays (dowels) under the conditions of forced convection^47^; cf. also the following works where 1D and 2D discrete fuel arrays were explored^48–52^. Still, a significant gap between the fundamental (massively interwoven with the empirical), and less fundamental (purely empirical) approaches, opens a niche for simplified models clearly elucidating some of the basic physical mechanisms responsible for forest flame propagation over hilly landscapes under windy conditions. Accordingly, the necessity and urgency of the present research aiming at spanning this gap. It should be emphasized that propagation of forest fire during the distillation stage is, in a sense, a particular example of the diffusion (non-premixed) flames, with volatiles (fuel) and oxidizer contained in the surrounding air being mostly separated and mixing and reacting only inside a narrow flame zone^9,53–57^. This situation is also typical for liquids, which, essentially, burn as a diffusion flame in vapor phase.

Broadly speaking, in his comprehensive review series Sullivan^58–60^ distinguished empirical models, quasi-empirical models, quasi-physical models, and physical models (practically all of them, CFD-based). The quasi-physical models do not delve into the combustion chemistry and, essentially, are supposed to treat the flame velocity as empirically given. It should be emphasized that the physical models aiming at predicting the flame velocity down to the chemistry involved, still involve massive pieces of empirical information regarding turbulence, the flame-turbulence interaction, the state and morphology of the forest and their effect on the flame propagation, etc. Using Sullivan’s classification, the theoretical/numerical model developed in the present work belongs to the class of quasi-physical models because it lumps the factors affecting the combustion rate (e.g., such as the type of trees, the volatile and moisture content in them^61^, the tree-to-tree distance and the overall pattern) into the normal velocity of flame propagation, which can be, in principle, measured in some separate experiments. With the normal velocity of flame propagation, the present model predicts the evolution of any initial given flame front based on solid physical principles, also accounting for the terrain and wind speed. In addition, Sullivan^58–60^ also emphasized that large-scale field experiments are costly and non-trivial to organize given multiple difficulties with wildfire recording and data processing, as well as the data interpretation, totality, and generalization (cf., for example, the large-scale experimental fires conducted across Corsican shrublands^62,63^, which makes lab-scale experiments attractive and illuminating at least on the qualitative level. This vision supports the lab-scale experimental approach also attempted in the present work.

This study investigates the effect of the following three key parameters—slope (inclination angle), wind presence, and inter-source spacing between flames—through controlled model experiments. Section  2 outlines the experimental setup and materials employed to replicate typical forest conditions, followed by the experimental results on flame propagation in the upslope and downslope directions under varying source spacings in Sect.  3. The combined influence of wind, slope, and source spacing on fire spread is systematically examined. A quasi-physical numerical model is introduced to predict forest flame-front propagation, first over flat terrain (Sect.  4) and subsequently over hilly landscapes (Sect.  5) under windy conditions. Representative results for each model are presented in Sects.  4 and 5, with conclusions drawn in Sect.  6. The latter is followed by ‘Perspectives and future work’ Sect.  7.

## Materials and experimental methods

**Fig. 1:**
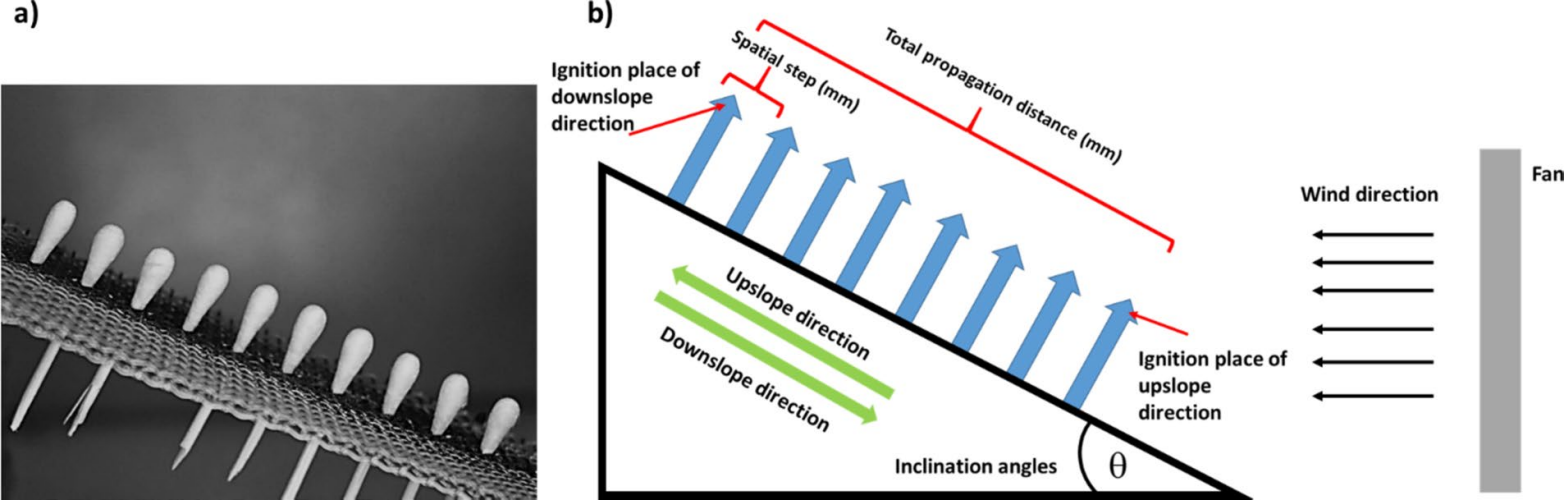
The flame propagation setup. (**a**) An image of a cotton-tipped applicator array, (**b**) illustration of the flame propagation setup.

The experimental setup is depicted in Fig. 1a. Fisherbrand cotton-tipped applicators (which will be designated as cotton from here on), pure or dipped in ethanol, were applied as the flame sources. A metallic mesh was used as a flame source holder. Cotton is located regularly and linearly over a surface which could be inclined at an angle θ or be horizontal (θ = 0^ο^). Ethanol 200 proof (100%) was purchased from Decon Laboratories, Inc. It should be emphasized that flammability of cotton resembles that of dry cellulose-based materials, such as dry bark and leaves, whereas ethanol is a realistic model of combustible volatiles contained in a number of plants and trees, e.g., in *Epipremnum aureum* (Golden Pothos) and an oak tree (*Quercus calliprinos*), and their vigorous eruptions in flame^9^. It should be emphasized that cotton tips were approximately of the same size and in cases they were pre-wetted, fully imbibed with ethanol. Accordingly, approximately the same amount of ethanol was soaked in each of them.

FLIR T1030sc IR camera and a DSLR camera (digital single-lens reflex camera, model Nikon D3100) were employed for recording the flame transport, with the former camera – at the acquisition rate of 30 Hz, and the latter one operated at 30 fps. The flame propagation morphologies and velocities were analyzed using NCH video editing software. In particular, this software allows one to extract locations of the leading point of the flame front pixel-wise during the entire time of flame propagation in all cases studied. A small fan located 10 cm away from the flame source was blowing along the linear array of cotton-tipped applicators (cf. Fig. 1b). Note that the fan was also used in the experiments in Ref^64^. to create wind acting on a simulated upslope and downslope fires. The wind speed was measured by HOLDPEAK 8668 digital anemometer. A quasi-steady state turbulent wind with a practically uniform longitudinal velocity profile characteristic of submerged turbulent jets was sustained in the present experiments^57,65^. The wind speed sustained in the 0.560 m/s to 0.734 m/s range at the location of 1 cm before the first cotton-tipped applicator revealed the turbulence intensity in the 9.46% to 14.80% range^65^. In the present work the wind speed generated by the fan was measured as 2.0 m/s. It should be emphasized that wind tunnels have also been used in the preceding work^66^, which revealed that flame propagation rate is highly sensitive to wind structure. The surrounding humidity was 16%. The experiments were conducted under room conditions, and no effect of the small day-to-day variations in temperature and humidity was observed.

The experiments were conducted with the two wind velocities, four spatial steps, at six different inclination angles, and for two spatial flame propagation distances listed in Table 1.

**Table 1:** The experimental settings.

Wind speed (m/s)	Spatial steps (mm)	Inclination angle (^ο^)	Spatial distance (mm)
0 or 2	9 11 13 15	0.0 13.5 22.3 31.0 40.4 53.9	95 100

## Experimental results and discussion

### The fire propagation in upslope / downslope direction with cotton dipped in ethanol as flame source with no wind effect

**Fig. 2:**
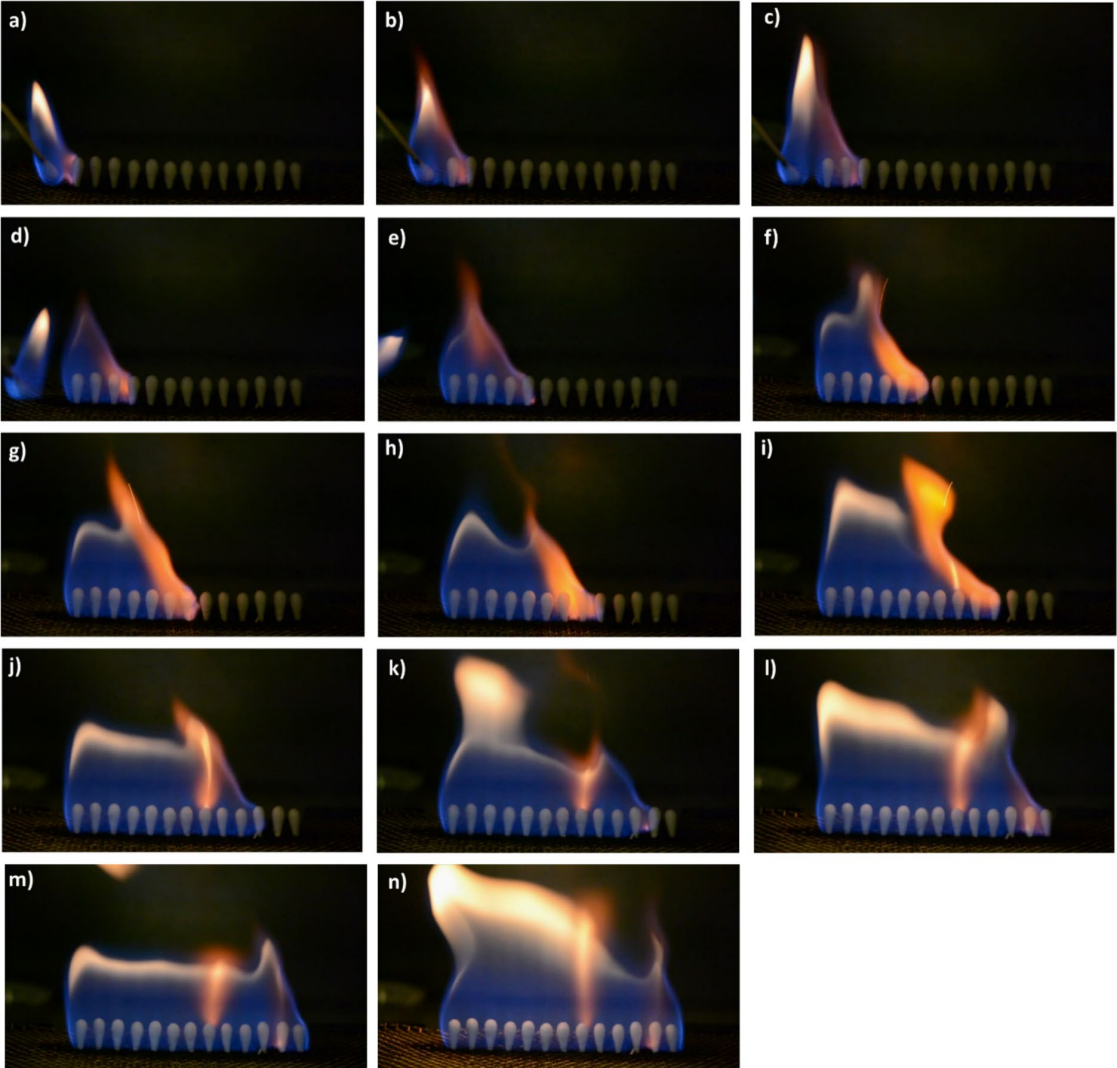
Flame propagation in the horizontal direction. Cotton dipped in ethanol serving as the flame source; recorded by the DSLR camera. The flame was not affected by the wind. The spatial step of this trial was 9 mm and the inclination angle was 0^ο^. (**a**) t = 0 s. (**b**) t = 0.066 s. (**c**) t = 0.132 s. (**d**) t = 0.198 s. (**e**) t = 0.264 s. (**f**) t = 0.330 s. (**g**) t = 0.396 s. (**h**) t = 0.462 s. (**i**) t = 0.528 s. (**j**) t = 0.594 s. (**k**) t = 0.660 s. (**l**) t = 0.726 s. (**m**) t = 0.792 s. (**n**) t = 0.858 s.

**Fig. 3:**
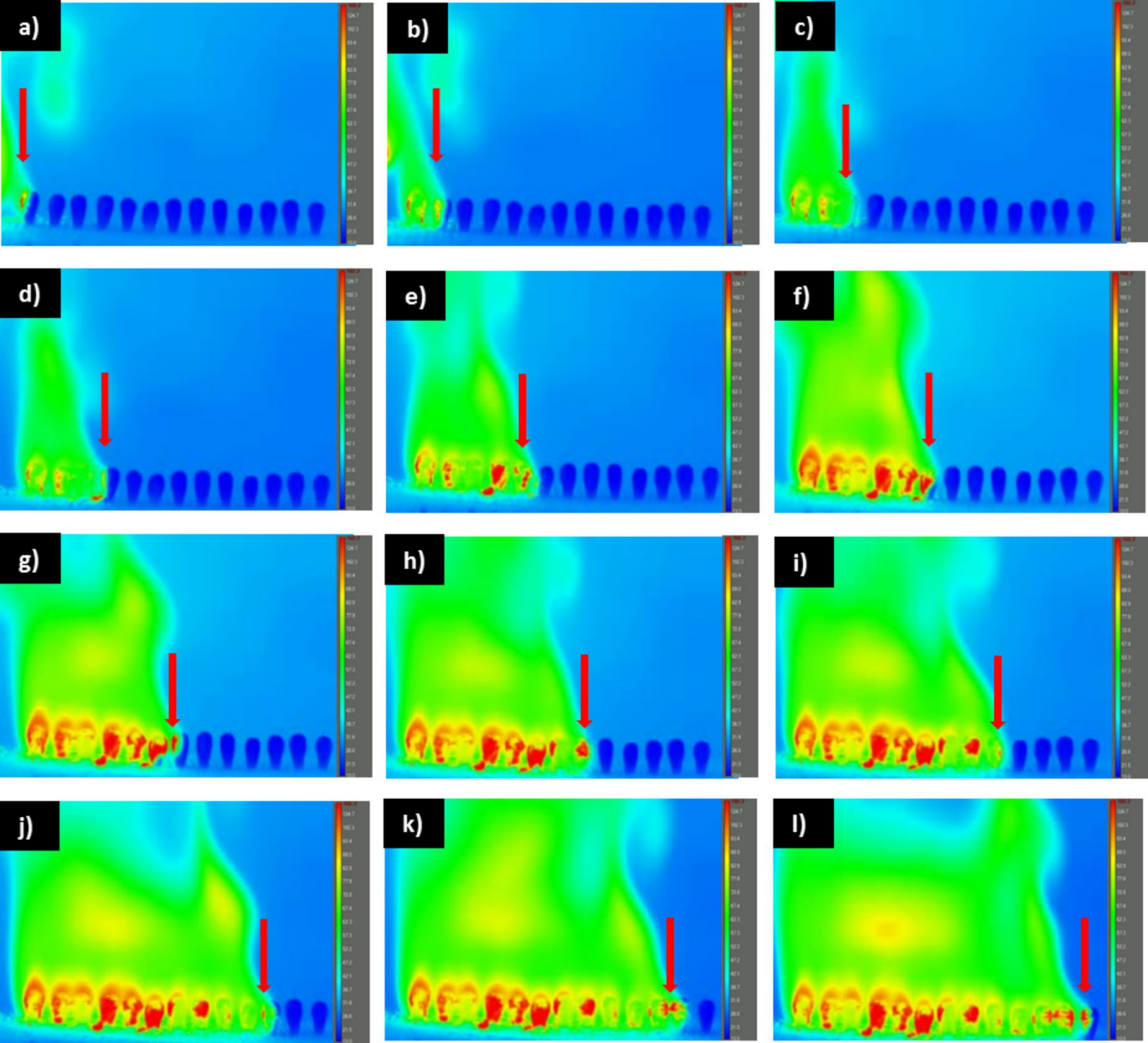
Flame propagation in the horizontal direction. Cotton dipped in ethanol serving as the flame source; recorded by the IR camera. The flame was not affected by the wind. The spatial step was 9 mm and the inclination angle was 0^ο^. (**a**) t = 0 s. (**b**) t = 0.066 s. (**c**) t = 0.132 s. (**d**) t = 0.198 s. (**e**) t = 0.264 s. (**f**) t = 0.330 s. (**g**) t = 0.396 s. (**h**) t = 0.462 s. (**i**) t = 0.528 s. (**j**) t = 0.594 s. (**k**) t = 0.660 s. (**l**) t = 0.726 s.

In this subsection, cotton dipped in ethanol were used as a flame source. Tests aiming flame transport were conducted thrice at each angle (cf. Table 1). Figure 2 presents the flame propagation progress in the trial of 9 mm spatial step with 0^ο^ inclination angles. Due to the high flammability of ethanol, the length of fully developed flame was the same as the total length of the flame source (cf. Fig. 2n). Propagation progress was shown much clearer by employing the IR camera. The appearance of the red zone at the surface of the flame source indicated the ethanol contained by cotton were ignited and the flame had propagated to this location (cf. Fig. 3). The color bars in infrared (IR) image sequences do not show a well-defined temperature due to the unknown emissivity of the burning ethanol contained in cotton. Accordingly, thermal images are used only to qualitatively track the front position. 

**Fig. 4:**
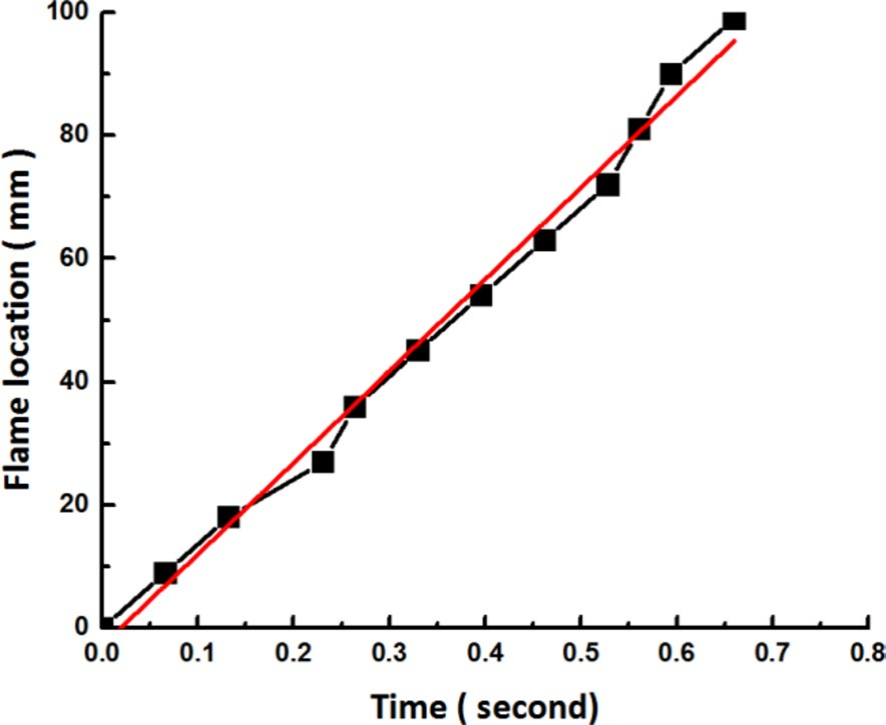
Flame propagation in the horizontal direction. Total propagation time versus propagation distance in the trial of 9 mm spatial step with the 0^ο^ inclination. Cotton dipped in ethanol served as a flame source. The red line indicated the fitting curve with the fitting equation ${\rm L= 148. 76t - 2.86}$. The experimental data presented is the arithmetic mean of three independent trials.

**Fig. 5:**
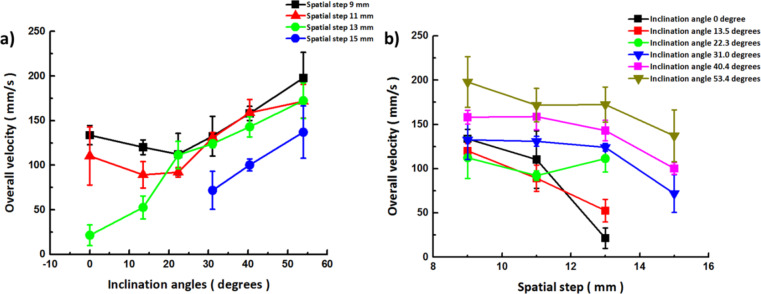
Flame propagation upslope. Measurement of the overall velocity using different spatial steps and inclination angles. The cotton dipped in ethanol was used as a flame source. (**a**) The overall velocity versus the inclination angle. (**b**) The overall velocity versus the spatial step. Here and hereinafter error bars illustrate standard error of the mean values. The experimental results are based on three independent trials for each spatial step and inclination angle.

Starting from the ignition moment of the first flame source to the moment of the last flame source had been ignited, the time for the fire to propagate through the array of flame sources was measured. One case of the experimental results was shown here as an example (cf. Fig. 4). In the trial of 9 mm spatial step with 0^ο^ of inclination, the flame propagated with nearly a constant velocity. The straight red line in Fig. 4 is the curve closely fitting the experimental data. The corresponding fitting equation was \rm L= 148. 76t - 2.86, where L is the total propagation distance and t is time. The slope of this equation was the overall velocity of the flame transportation. This measurement criteria and calculating methods were applied in all experiment trials. The average values of the overall velocities are shown in Fig. 5.

The fire could successfully transport in upslope direction through the flame source with the 9 mm, 11 mm, and 13 mm spatial steps at all inclination angles, whereas it failed to transport with the 15 mm spatial steps at the inclination angles 0^ο^, 13.5^ο^, and 22.3^ο^. It should be emphasized that the size of the ignition zone of the second cotton-tipped applicator behind the first burning cotton-tipped applicator is limited by heat transfer, and an increase in the spatial step size ultimately prevents fire transport, like a tree-to-tree flame propagation explored in detail experimentally and theoretically in the previous work of this group in Ref^57^.

The flash point of pure ethanol is about 17 °C and the maximum value of the overall transport velocity obtained in these experiments was 230.41 mm/s, which was obtained from the test of 9 mm spatial steps with 53.9^ο^ inclination angle. When the inclination angles were smaller than 22.3 ^ο^, the overall velocities of fire transport in the flame source with 9 mm and 11 mm spatial steps slightly decreased (or rather plateaued, given the error bars), and the values obtained from the 9 mm spatial step experiments were larger than those from the 11 mm experiments. The plateauing mentioned above presumably means that the buoyancy-associated pre-heating accelerating flame propagation upslope is still not felt at such small inclination angles. When the inclination angle was larger than 22.3^ο^, the overall velocity values obtained in the trials of 9 mm were nearly the same as the ones obtained from the corresponding experiments with 11 mm and 13 mm, i.e., in these three groups of experiments, the effect from the spatial steps on the flame transport velocity was not significant when the material was highly flammable and the inclination angles were larger than 30 ^ο^.

**Fig. 6:**
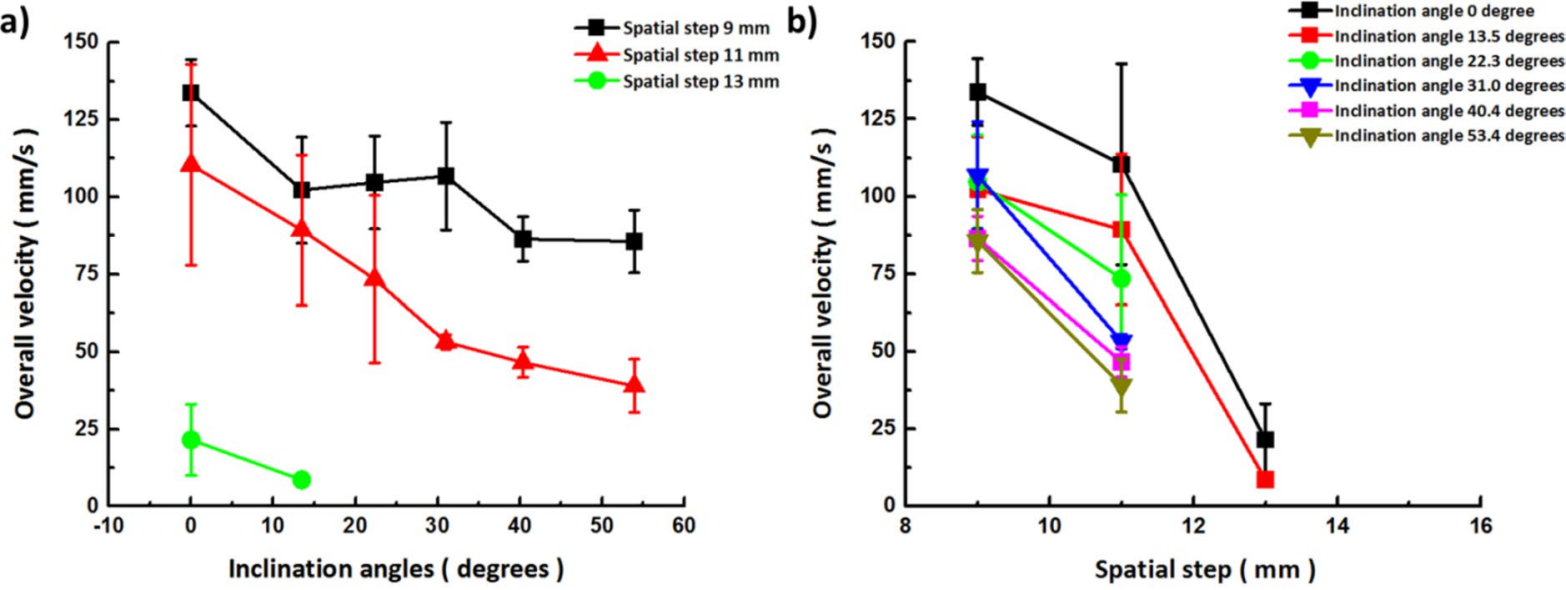
Flame propagation downslope. The measured overall velocity with various spatial steps and inclination angles. The cotton dipped in ethanol was used as a flame source. The fire propagated in the downslope direction. (**a**) The overall velocity versus the inclination angle. (**b**) The overall velocity versus the spatial step. The experimental data are based on the arithmetic means of three independent trials for each spatial step and inclination angle.

The fire was successfully propagated downslope through the flame source with the 9 mm and 11 mm spatial steps at all inclination angles. As the spatial step was 13 mm, the flame could only transport in the downslope direction with the inclination angles 0^ο^ and 13.5^ο^. Here and hereinafter the downslope inclination angle values mean their magnitudes. Experiments with a spatial step of 15 mm failed to propagate fire. The maximum value of the overall transport velocity obtained in these tests was 145.78 mm/s, which was obtained from the test with the 9 mm spatial steps at 0^ο^ inclination angle. With the increasing inclination angle magnitude, the velocity of fire transport through the flame source predominantly decreased (cf. Fig. 6a). A slight increase in the flame velocity at the spatial step of 9 mm close to the downslope angle of 30º might be caused by relatively large error bars there, which can mask a gradual decrease. In the downslope experiments, the smallest spatial step yielded the highest overall propagation velocities relative to the corresponding larger-step tests, indicating a significant spacing effect (Fig. 6).

### The fire propagation in the upslope / downslope direction with pure cotton as the flame source with no wind effect

**Fig. 7:**
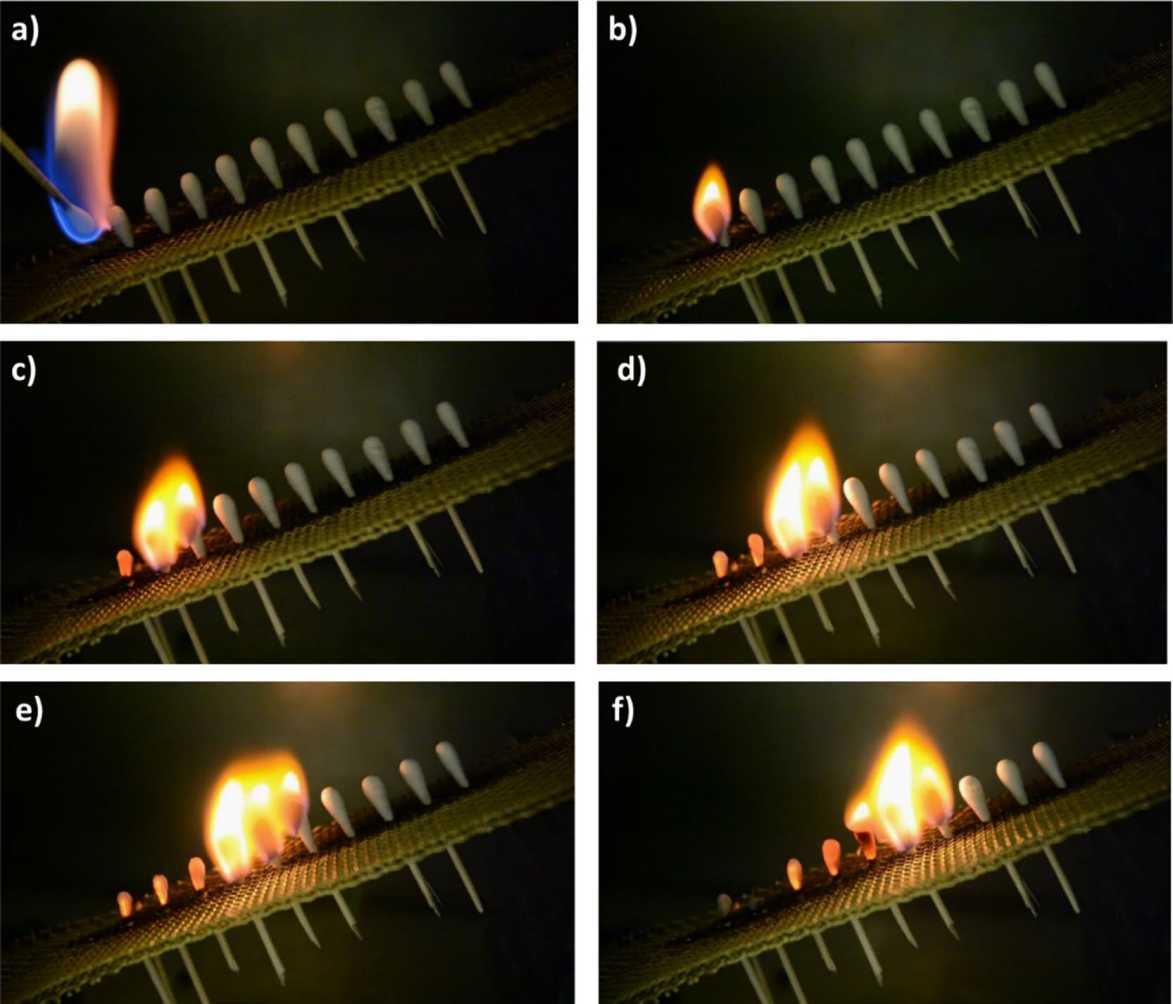
Flame propagation upslope. The progress of flame propagating in pure cotton as a flame source, recorded by the DSLR camera. The flame was not affected by the wind. The spatial step of this trial was 11 mm, and the inclination angle was 40.0^ο^. (**a**) t = 0 s. (**b**) t = 5 s. (**c**) t = 25 s. (**d**) t = 35 s. (**e**) t = 45 s. (**f**) t = 55 s.

**Fig. 8:**
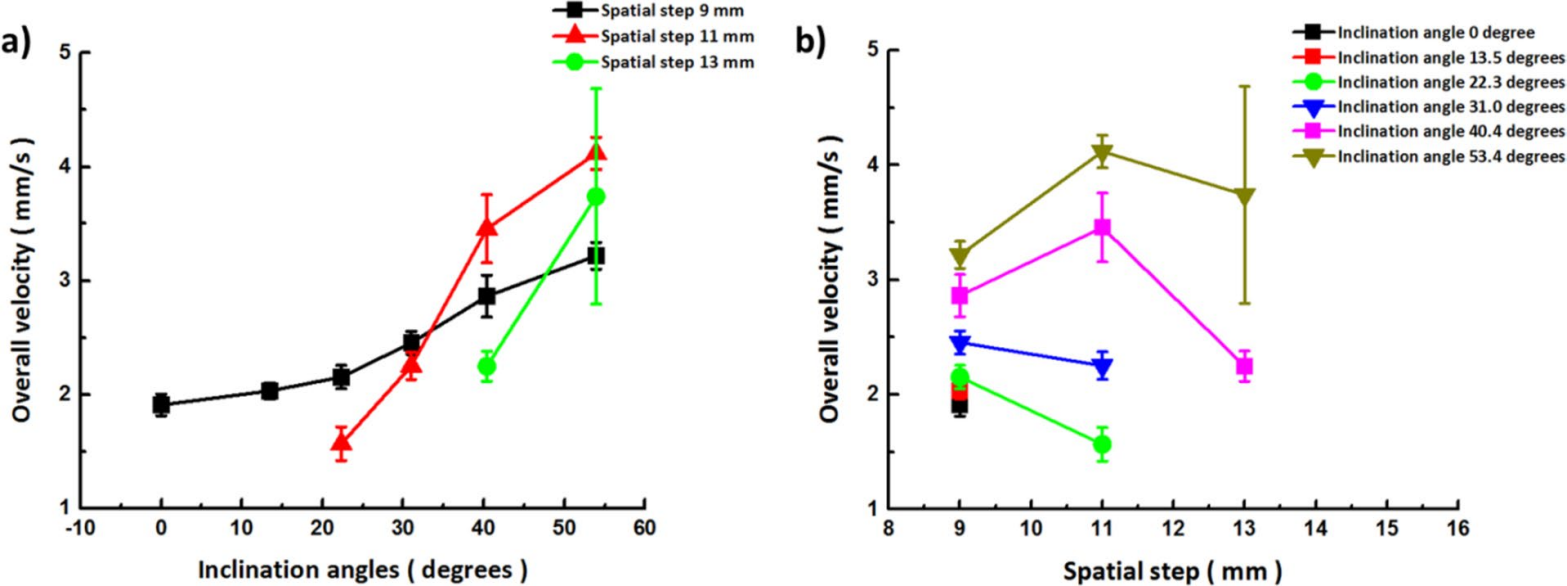
Flame propagation upslope. The measured overall velocity with various spatial steps and inclination angles. The pure cotton was used as a flame source. The flame propagated in the upslope direction. (**a**) The overall velocity versus the inclination angle. (**b**) The overall velocity versus the spatial step. The experimental results are the arithmetic mean of three independent trials for each spatial step and inclination angle.

**Fig. 9:**
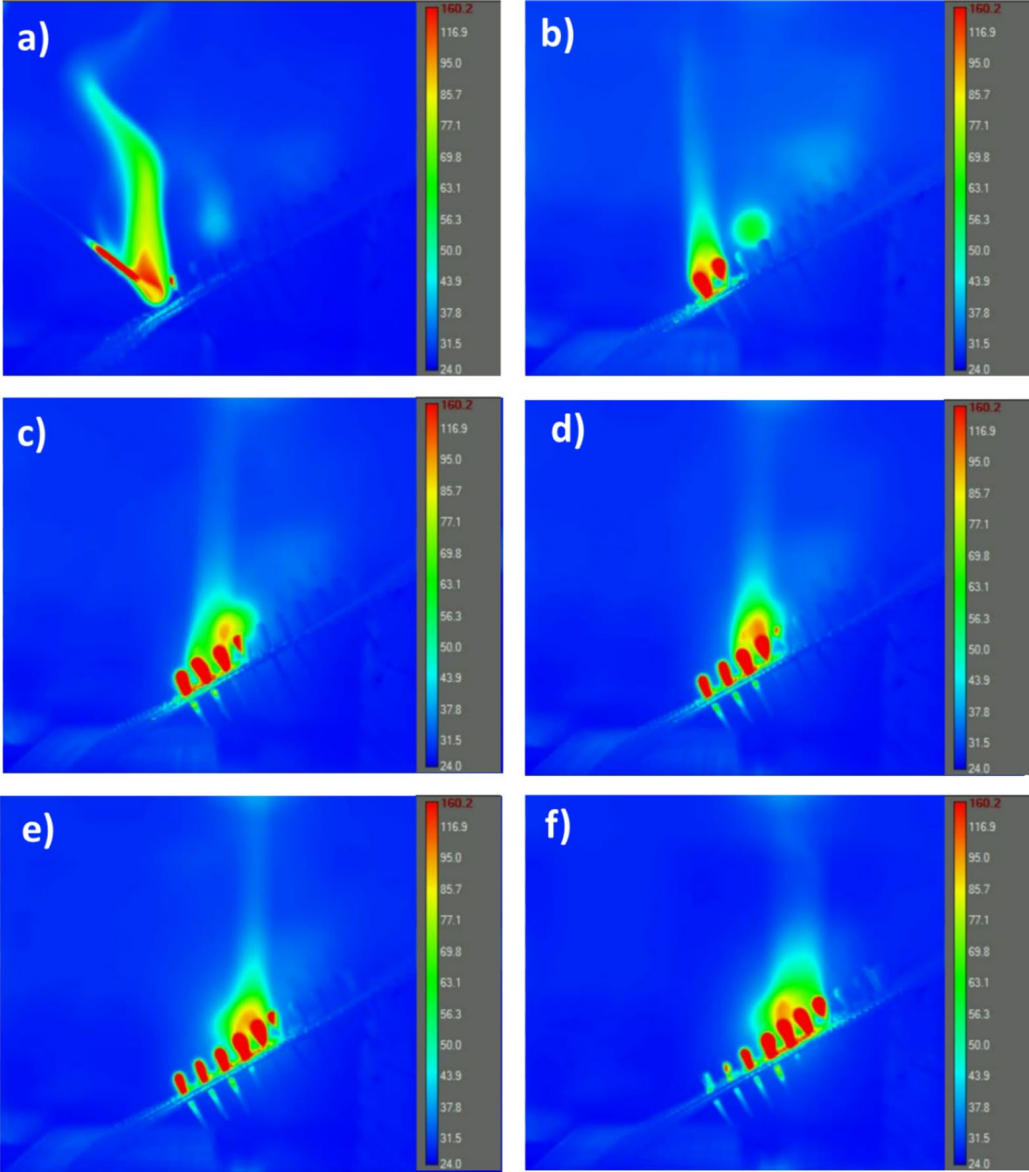
Flame propagation upslope. The progress of flame propagating in pure cotton as a flame source, recorded by the IR camera. The flame was not affected by the wind. The spatial step of this trial was 11 mm, and the inclination angle was 40.0^ο^ (**a**) t = 0 s. (**b**) t = 5 s. (**c**) t = 25 s. (**d**) t = 35 s. (**e**) t = 45 s. (**f**) t = 55 s.

In this subsection, experiments were conducted with the pure cotton. The experimental setup, measurement methods, and the judging criteria used here were the same as those in the previous subsection. In the previous subsection, at the end of the observation the flame length was equal to the entire length of the flame source due to the high flammability of ethanol, and the cotton would not start to burn until the ethanol was burned out. Thus, the cotton tips remained intact and white after the experiments when fire spread all over the source, i.e., the tips stayed white and uncharred in the images recorded by the DSLR camera (cf. Fig. 2n). The flame transport observed in the present subsection (cf. Fig. 7e-f) was different from the previous subsection. After igniting the flame source, the cotton was charred, which is clearly seen in Fig. 7f, and the flame slowly spread to the surface until the entire flame source was surrounded by the fire. The flame source after the experiments became ashes. The fully developed flame length found here was about 2 cm. The experimental results obtained are presented in Figs. 7, 8   and 9. The color bars in the infrared (IR) image sequences do not show a well-defined temperature due to the unknown emissivity of the burning cotton.

The ignition temperature of the cotton is 210 °C and the cotton must be ignited by an open flame for more than 3 s. The flame was successfully propagated in the upslope direction in the experiments at the spatial step 9 mm with all inclination angles, whereas it failed to transport at the 11 mm spatial step at 0^ο^ or 13.5^ο^ inclination angles and the 13 mm spatial step at 0^ο^, 13.5^ο^, 22.3^ο^ and 31.0^ο^. The fire could not be transported at any inclination angles in the 15 mm spatial step experiments. As illustrated in Fig. 8a, the flame transport velocity would increase with the increasing inclination angles. The maximum overall velocity obtained here was 4.27 mm/s, which was much smaller than the corresponding value obtained in the previous subsection. When the inclination angles were larger than 40.4 ^ο^, the changing rates of the overall velocity for the 9 mm, 11 mm, and 13 mm spatial steps were calculated as 0.026 mm/ s degree, 0.048 mm/ s degree, and 0.110 mm/ s degree. Accordingly, the increasing values of spatial steps (within the limits that fire could prapogate) will accelerate the flame velocity if the flame source is not highly flammable.

**Fig. 10:**
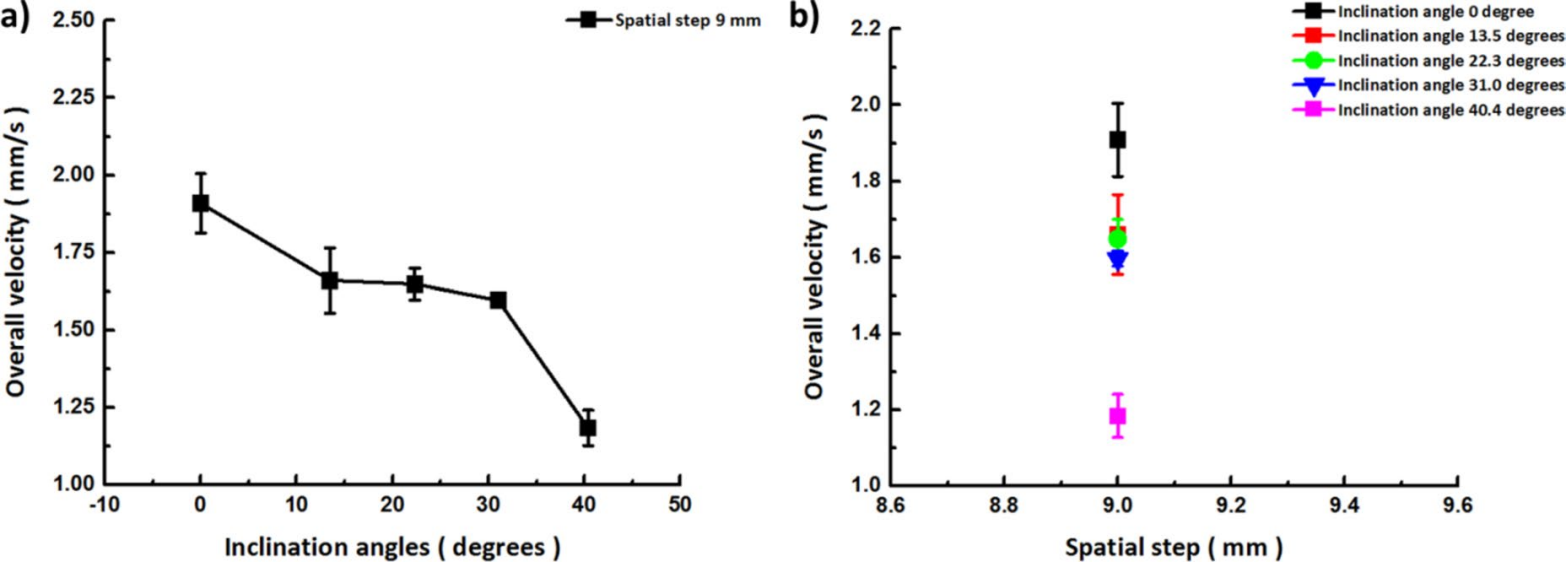
Flame propagation downslope. The measured overall velocity with various spatial steps and inclination angles. The pure cotton was used as a flame source. The flame propagated in the downslope direction. (**a**) The overall velocity versus the inclination angle. (**b**) The overall velocity versus the spatial step. The experimental results are the arithmetic mean of three independent trials for each spatial step and inclination angle.

The flame propagated downslope only in the experiments with the spatial step of 9 mm, across all inclination angles. The pure cotton was used as a flame source. The measured overall velocity at various spatial steps and inclination angles is shown in Fig. 10. The velocity decreased with the increasing inclination angles and the maximum value of the overall velocity obtained here was 2.02 mm/s, which corresponds to the trial with the 9 mm spatial step at the 0^ο^ inclination angle. The downslope flame propagation significantly diminishes the conductive, convective, and radiative heat fluxes toward the unburnt material, which is seemingly the main reason for slowing down the flame propagation rate as the magnitude of the inclination angle increases. The character of the descending dependence revealed in Fig. 10a is non-monotonous and the decrease in the flame velocity above ~ 30^ο^ becomes, essentially, catastrophic, preceding a complete quenching. The diminishing heat fluxes also suffocate flame propagation over the arrays with spatial steps larger than 9 mm.

### The flame propagation in the upslope / downslope direction with pure cotton as the flame source with wind effect

**Fig. 11:**
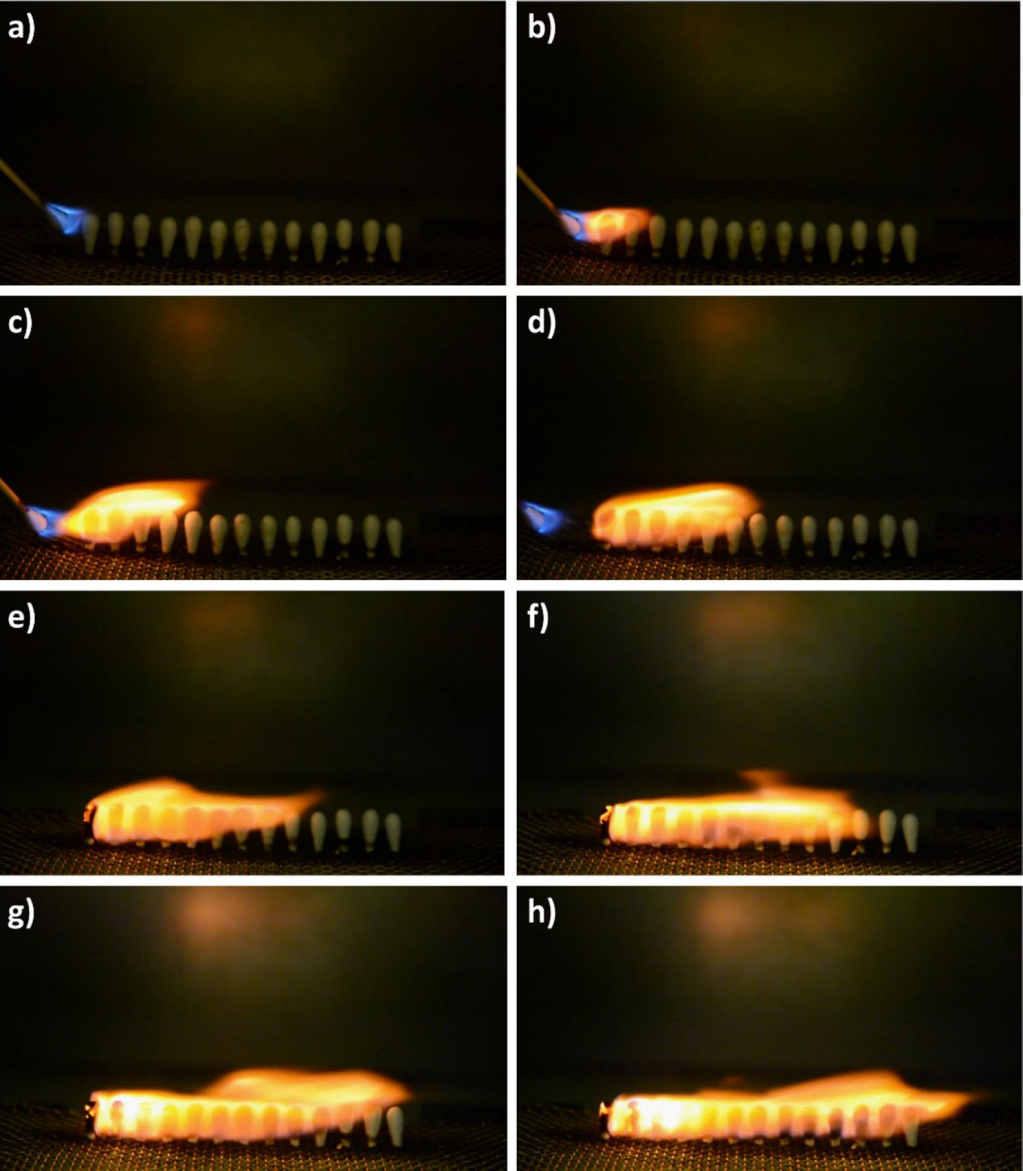
Flame propagation in the horizontal direction. The progress of flame propagating in pure cotton as a flame source, recorded by the DSLR camera. The flame was affected by the 2.0 m/s horizontal wind. The spatial step of this trial was 9 mm and the inclination angle was 0^ο^. (**a**) t = 0 s. (**b**) t = 2 s. (**c**) t = 4 s. (**d**) t = 6 s. (**e**) t = 8 s. (**f**) t = 10 s. (**g**) t = 12 s. (**h**) t = 14 s.

**Fig. 12:**
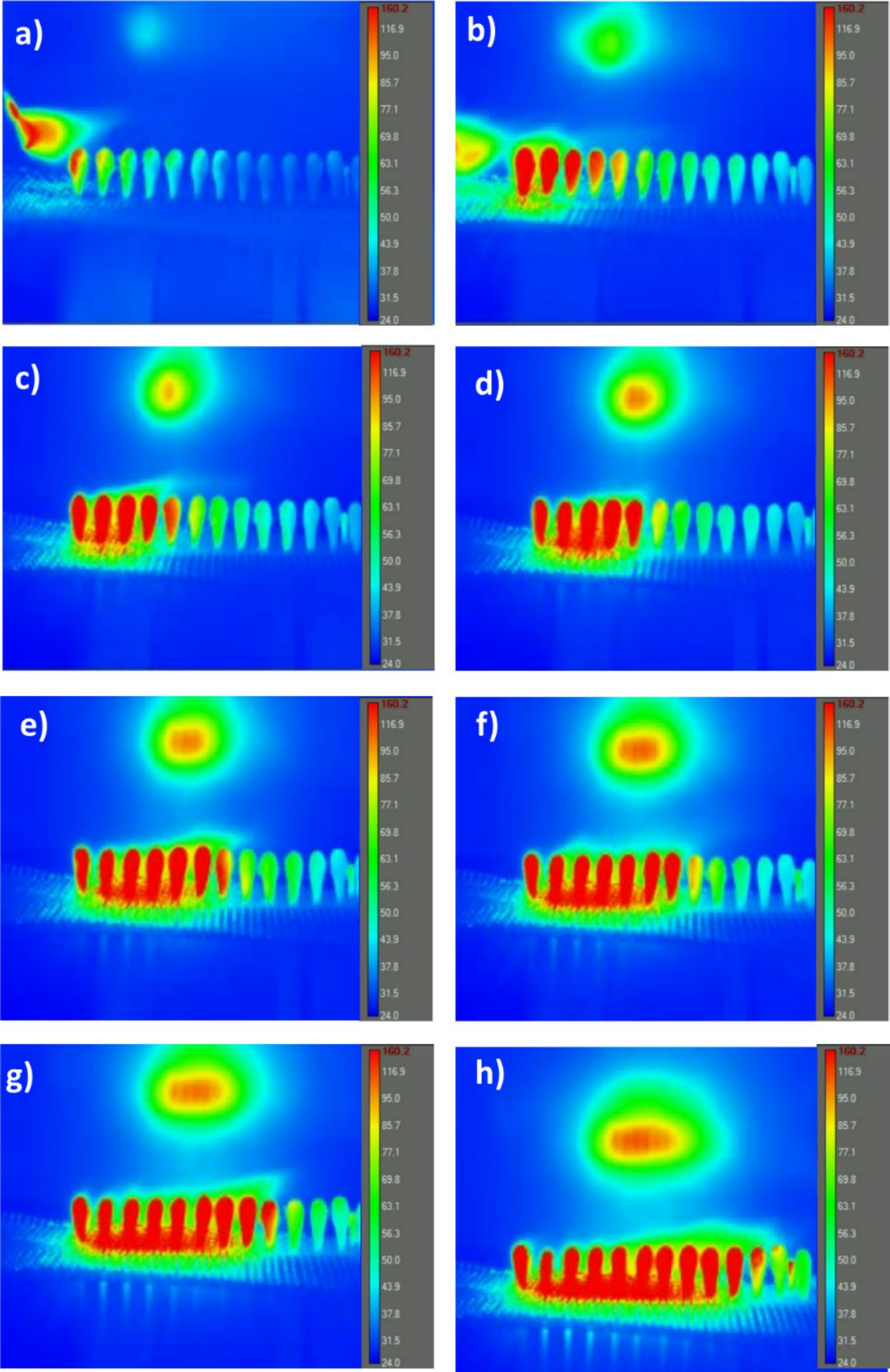
Flame propagation in the horizontal direction. The progress of flame propagating in pure cotton as a flame source, recorded by the IR camera. The flame was affected by the 2.0 m/s horizontal wind. The spatial step of this trial was 9 mm, and the inclination angle was 0^ο^. (**a**) t = 0 s. (**b**) t = 2 s. (**c**) t = 4 s. (**d**) t = 6 s. (**e**) t = 8 s. (**f**) t = 10 s. (**g**) t = 12 s. (**h**) t = 14 s.

In this subsection, the effect of the wind on the flame velocity, as well as the inclination angle effect and the spatial steps effect, were studied. The experiments were conducted with pure cotton as flame sources. The wind speed generated from the fan was measured as 2.0 m/s. The flame propagation progress was recorded by the DSLR camera (cf. Fig. 11) and the IR camera (cf. Fig. 12).

**Fig. 13:**
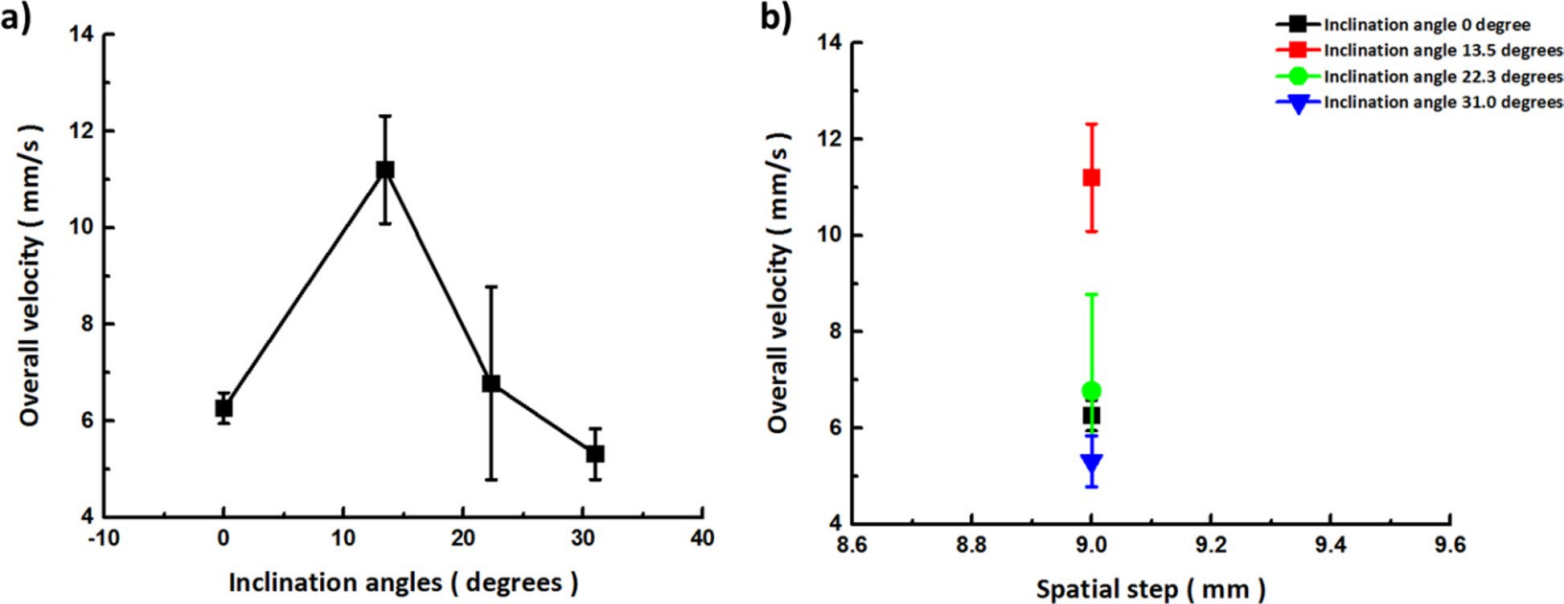
Flame propagation upslope. The measured overall velocity with various spatial steps, inclination angles, and the 2 m/s wind. The pure cotton was used as a flame source. The fire propagated in the upslope direction. (**a**) The overall velocity versus the inclination angle. (**b**) The overall velocity versus the spatial step. The experimental results are the arithmetic mean of three independent trials for each spatial step and inclination angle.

With the horizontal wind (cf. Fig. 1b), the flame only propagated in the upslope direction in the experiments of spatial step 9 mm with 0^ο^, 13.5^ο^, 22.3^ο^, 31.0^ο^, 40.4^ο^ angles. The measured overall velocity with various spatial steps and inclination angles is shown in Fig. 13. When the inclination angles changed from 0^ο^ to 13.5^ο^, the existence of wind and increased inclination angles contributed to an increased overall flame velocity. The maximum value of the overall velocity found was 12.49 mm/s, which was obtained from the trial with the 13.5^ο^ inclination angle and was larger than the corresponding values shown in the previoud subsection. When the inclination angle was equal to 0^ο^, the average velocity values of the fire propagation were measured as 6.26 mm/s, which was increased 3.3 times by the 2 m/s wind compared with the corresponding value (1.91 mm/s) obtained in the experiments with no wind effect. When the inclination angle was equal to 13.5^ο^ the average velocity value of the fire propagation was increased 5.5 times by the 2 m/s wind compared with the corresponding value (2.03 mm/s) in the previous subsection.

As the inclination angles continued increasing, the fire continuously propagated over the source. Thus, the flame could only propagate through the bottom of the cotton in the upslope direction, which significantly decreased its velocity. When the inclination angle was changed to 40.4 ^ο^, the flame failed to propagate. The obtained experimental results are shown in Fig. 13. Also, the fire could not propagate in the downslope direction when wind existed (The wind direction was the same as before).

## Model development: planar flame fronts

The model developed in the present and the following sections implies that the flame propagation is sustained by volatiles rapidly released by plants in fire, corresponding to the eruptive volatile release observed experimentally in Ref^9^. Accordingly, the volatiles serve as fuel sustaining propagating flame, which is characteristic of propagation of forest fire during the distillation stage. The volatiles mix with oxidizer from the ambient air in a thin flame front like in diffusion flames of liquids vapor burns over the liquid surface rather than the liquid phase itself. The model also implies that the flame front can be considered as a continuous line on the entire fire length scale, which is much larger than the tree-to-tree distances. The latter means mathematically that the so-called diffusion approximation is used similarly to diffusion processes invoked for Markovian processes, which are reduced to continuous paths at a large number of steps^67,68^, or to the statistical theory of polymer macromolecules, when the Fokker-Planck equation governing a continuous probability-density function is used for the large number of Kuhn segments^67,69,70^, or to the renormalization group theory of calender-bonded composite nonwoven materials^71^.

The present work deals with the distillation phase of forest fires, when volatiles erupting from stomata in leaves and stems due to heating from the surrounding flame serve as the fuels sustaining the combustion process in the flame, rather than solid and porous structures themselves. This is similar to burning liquids and weapon powders, whereas the wind-entrained turbulent flame is in a sense similar to the so-called free turbulence flows in submerged gaseous jets and torches^53,65^. Consider first the propagation of a flame front over a plane. Introduce radial and azimuthal polar coordinates on the plane, r and φ, respectively. The radius-vector of the flame front $\mathbf{R}\left( {t,\varphi } \right)$is

4.1 $$\mathbf{R}\left( {t,\varphi } \right)=R\left( {t,\varphi } \right){\mathbf{e}_r}\left( \varphi \right)$$

where $R\left( {t,\varphi } \right)$is its magnitude, ${\mathbf{e}_r}\left( \varphi \right)$is the unit vector of the radial direction, and t is time.

The outer unit normal vector **n** to the flame front is found, accordingly, as

4.2 $$\mathbf{n}=\frac{{{\mathbf{e}_r} - {\mathbf{e}_\varphi }{R^{ - 1}}\partial R/\partial \varphi }}{{\sqrt {1+{R^{ - 2}}{{\left( {\partial R/\partial \varphi } \right)}^2}} }}$$

where ${\mathbf{e}_\varphi }\left( \varphi \right)$ is the unit vector of the azimuthal direction.

According to Eq. (4.2) the angle α between the normal to the flame front **n** and the radial direction determined by the unit vector ${\mathbf{e}_r}$ is found as

4.3 $$\cos \alpha =\mathbf{n}\cdot {\mathbf{e}_r}=\frac{1}{{\sqrt {1+{R^{ - 2}}{{\left( {\partial R/\partial \varphi } \right)}^2}} }}$$

Denote the normal velocity of flame propagation as V_n_. It should be emphasized that the expected thermal range corresponding to the distillation stage of the forest fires is sufficient for vigorous eruptions of volatiles from biomass^57^, whereas the physical mechanism of the flame propagation (thermal or thermal and radiative) is lumped in the normal velocity of flame propagation V_n_ in the present model.

Then, the equation of flame propagation is established as

4.4 $$\frac{{\partial R}}{{\partial t}}\cos \alpha ={V_n}$$

which yields the following equation determining the configuration of the flame front on the plane in time

4.5 $$\frac{{\partial R}}{{\partial t}}={V_n}\sqrt {1+{R^{ - 2}}{{\left( {\partial R/\partial \varphi } \right)}^2}}$$

This is, essentially, a particular case of the eikonal equation. The solution is subjected to the following initial condition

4.6 $$t=0,\quad R={R^0}\left( \varphi \right)$$

where the function ${R^0}\left( \varphi \right)$ is determined by the configuration of an initial ignition area, and can be taken, for example, as a perturbed circle

4.7 $${R^0}\left( \varphi \right)={R_0}\left( {1+\varepsilon \sin n\varphi } \right)$$

In Eq. (4.7) R_0_ is the circle radius, ε and n are the dimensionless perturbation amplitude and the azimuthal wavenumber, respectively.

It should be emphasized that the velocity of flame front propagation on a plane depends on its curvature as^54–56^

4.8 $${V_n}={V_{n0}}\left( {1 - {L_\mu }k} \right)$$

where V_n0_ is the velocity of propagation of a straight flame front, L_µ_ is the characteristic Markstein length, and k is the curvature of the flame front (in the differential geometry, mathematical sense), which is given in the particular case by the following formula^72^

4.9 $$k=\frac{{{R^2}+2{{\left( {\partial R/\partial \varphi } \right)}^2} - R\left( {{\partial ^2}R/\partial {\varphi ^2}} \right)}}{{{{\left[ {{R^2}+{{\left( {\partial R/\partial \varphi } \right)}^2}} \right]}^{3/2}}}}$$

It should be emphasized that the present model is geometric in nature and lumps all physical phenomena affecting flame propagation into the velocity of flame front propagation V_n_ including the Markstein correction similarly to Ref^73^., which considered wildfire fronts. The difference with Ref^73^. here is in the fact that the present model directly employs the geometric fact that any front (flame, in the present case, or light, or sound, etc.) propagating with a normal velocity V_n_ is supposed to develop fingering because its propagation is governed by the eikonal Eq. (4.5). This fact is well known in the classical combustion theory^54^. It should be emphasized that any tendency to the short-wavelength fingering can be suppressed by the Markstein correction, which serves in the present context as a regularization factor accounting for two physical stabilizing mechanisms: the concave section of the flame front converges heat flux, overheats itself and thus accelerates and straightens, whereas the convex section diverges the heat flux and thus decelerates and once again, straightens. On the other hand, Ref^73^. introduces an additional source of flame instability related to suction of oxygen from the ambient air associated with ‘some constant’, as per Ref^73^. The latter mechanism can be attributed to the diffusional-thermal instability of flame^54^ and can also be suppressed by the Markstein correction^73^. Moreover, one can also mention the Darrieus-Landau aerodynamic flame instability^54^ which can also be suppressed by the Markstein correction, albeit both the diffusional-thermal instability of flame and the aerodynamic flame instability imply a significant pre-mixing of fuel and oxidizer uncharacteristic for fire fronts at the distillation stage of forest fire.

Render the problem dimensionless using R_0_ as the scale for R, $R_{0}^{{ - 1}}$ as the scale for k, and R_0_/V_n0_ as the scale for t, and drop the overbars over the dimensionless variables for brevity. Then, the dimensionless problem takes the following form

4.10 $$\frac{{\partial R}}{{\partial t}}=\left( {1 - \mu k} \right)\sqrt {1+{R^{ - 2}}{{\left( {\partial R/\partial \varphi } \right)}^2}}$$

4.11 $$t=0,\quad R=1+\varepsilon \sin n\varphi$$

where the dimensionless group µ = L_µ_/R_0_, and the curvature k is given by Eq. (4.9).

**Fig. 14:**
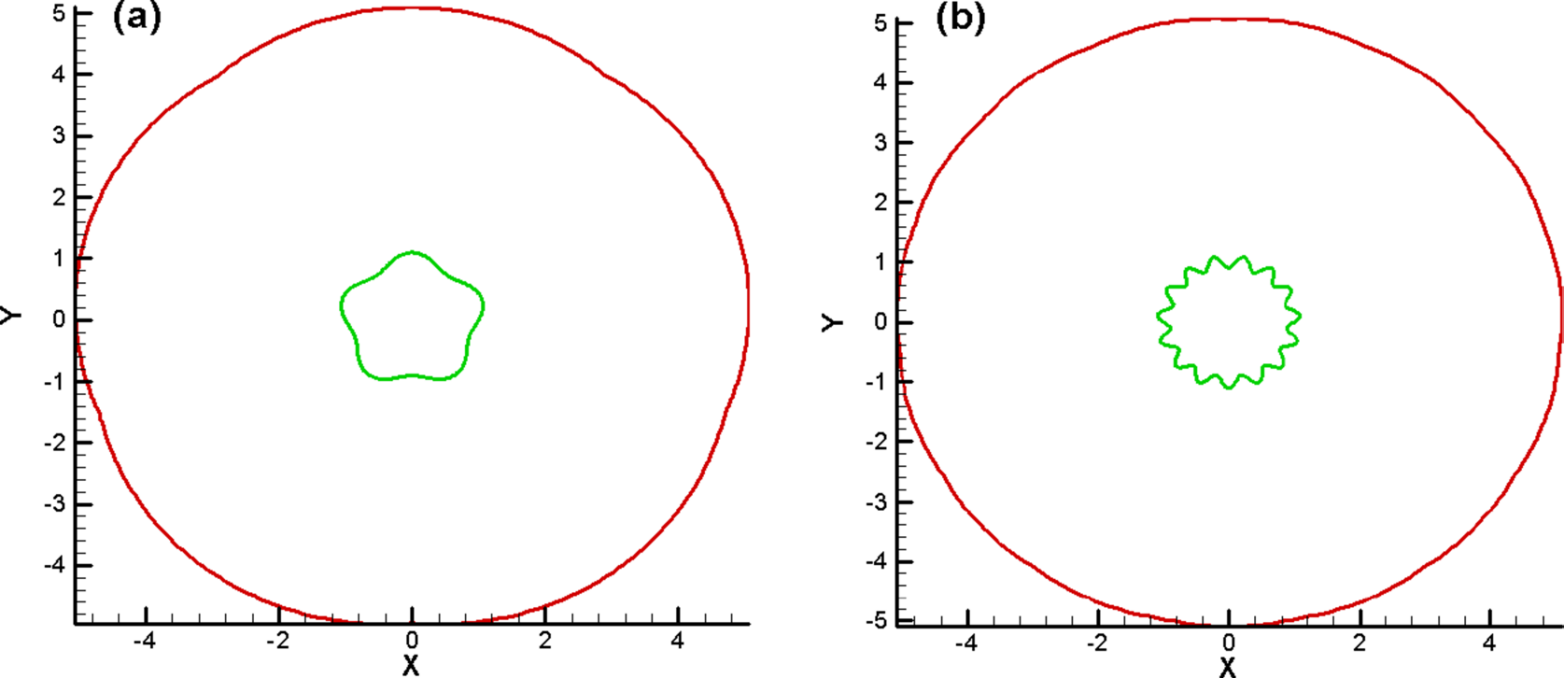
Flame front propagation on a plane. The green curves show the initial frame front at t = 0, the red lines show the flame front at t = 4. The ignition area is centered at X = Y=0, with X and Y being the Cartesian coordinates on the plane. (**a**) The wavenumber of the initial perturbation *n* = 5, and the dimensionless perturbation amplitude ε = 0.1. (**b**) The wavenumber of the initial perturbation *n* = 15, the dimensionless perturbation amplitude ε = 0.1, and $\mu ={10^{ - 3}}$.

The problem (4.9)-(4.11) is solved numerically and two examples of the solutions describing flame propagation on a plane from an ignition area are shown in Fig. 14. It is seen that the flame propagating on a plane readily stabilizes the initial perturbations of a circle and becomes almost circular at distances large enough from the ignition center. This happens due to the fact that the heat-up of the fuel (forest) ahead of a concave section of the flame front is enhanced by the converging heat flux from the flame front, and thus, the flame accelerates at this section and “catches up”. On the other hand, the heat-up of the fuel (forest) ahead of a convex section of the flame front is diminished due to the diverging heat flux from the flame front, and thus flame decelerates at this section and “lines up”. The above-mentioned physical phenomena are expressed by the Markstein formula (4.8) and its implementation in Eq. (4.10) and its solutions in Fig. 14.

## Three-dimensional flame fronts propagating over a hilly landscape

Consider now a flame front propagation in a forest on a hilly landscape z = f(x, y), where x, y and z are the Cartesian coordinates and f(x, y) is an arbitrary function expressing the landscape altitudes. Accordingly, the outer normal to the landscape surface **N** is

5.1 $$\mathbf{N}=\frac{{ - \mathbf{i}\partial f/\partial x - \mathbf{j}\partial f/\partial y+\mathbf{k}}}{{\sqrt {1+{{\left( {\partial f/\partial x} \right)}^2}+{{\left( {\partial f/\partial y} \right)}^2}} }}$$

where **i**, **j** and **k** are the unit vectors of the coordinate axes x, y and z, respectively.

**Fig. 15:**
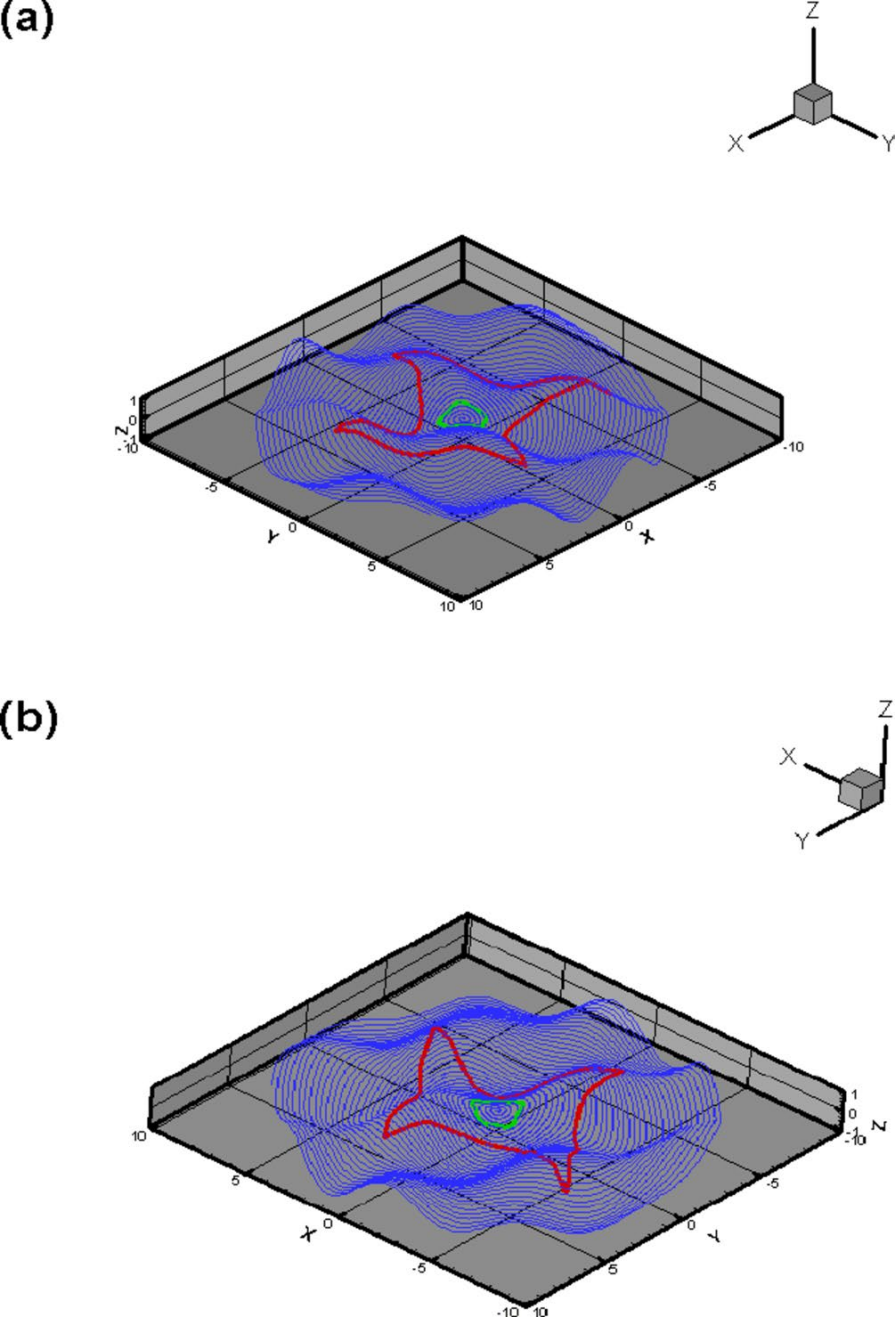
Flame front propagating over the hilly landscape shown in two perspectives (**a**) and (**b**). Green lines show the initial flame front at t = 0 (the ignition area is encircled by this flame front). Red lines show the flame front at t = 4. The spiraling blue lines centered at the ignition center are used to visualize the landscape. The wavenumber of the initial perturbation *n* = 5, the dimensionless perturbation amplitude ε = 0.1, and $\mu ={10^{ - 3}}$. The dimensionless wavenumbers are K_x_=1 and K_y_=0.5, and the ratio R_0_/z_0_ = 1. The wind velocity is zero, i.e., U_x_=U_y_=0.

It should be emphasized that the Cartesian coordinates x and y (as well as the polar coordinates R and φ) belong to the terrain foundation, whereas z is the coordinate reckoned from the foundation as an elevation, similarly to an altitude over sea level (cf. Fig. 15). In the present case, the radius-vector of the flame front at any time moment can still be expressed single-parametrically using the polar coordinates introduced before as

5.2 $$\mathbf{R}=R\left( {t,\varphi } \right){\mathbf{e}_r}\left( \varphi \right)+\mathbf{k}f\left[ {R\left( {t,\varphi } \right)\cos \varphi ,\;R\left( {t,\varphi } \right)\sin \varphi } \right]$$

which is convenient.

Then, the unit tangent vector to the flame front **τ** is found, accordingly as

5.3 $$\begin{gathered} \boldsymbol{\uptau}=\{ \mathbf{i}\left( { - \sin \varphi +\frac{1}{R}\frac{{\partial R}}{{\partial \varphi }}\cos \varphi } \right)+\mathbf{j}\left( {\cos \varphi +\frac{1}{R}\frac{{\partial R}}{{\partial \varphi }}\sin \varphi } \right) \hfill \\ \quad \quad +\mathbf{k}\left[ {\frac{{\partial f}}{{\partial x}}\left( {\frac{1}{R}\frac{{\partial R}}{{\partial \varphi }}\cos \varphi - \sin \varphi } \right)+\frac{{\partial f}}{{\partial y}}\left( {\frac{1}{R}\frac{{\partial R}}{{\partial \varphi }}\sin \varphi +\cos \varphi } \right)} \right]\} \hfill \\ \quad \quad /{\{ 1+\frac{1}{{{R^2}}}{\left( {\frac{{\partial R}}{{\partial \varphi }}} \right)^2}+{\left[ {\frac{{\partial f}}{{\partial x}}\left( {\frac{1}{R}\frac{{\partial R}}{{\partial \varphi }}\cos \varphi - \sin \varphi } \right)+\frac{{\partial f}}{{\partial y}}\left( {\frac{1}{R}\frac{{\partial R}}{{\partial \varphi }}\sin \varphi +\cos \varphi } \right)} \right]^2}\} ^{^{{^{{^{{^{{^{{1/2}}}}}}}}}}}} \hfill \\ \end{gathered}$$

Then, the outer normal to the flame front **n** is readily found as the vector product $\mathbf{n}=\boldsymbol{\uptau} \times \mathbf{N}$, which yields

5.4 $$\mathbf{n}=\mathbf{i}\left( {{\tau _y}{N_z} - {\tau _z}{N_y}} \right)+\mathbf{j}\left( {{\tau _z}{N_x} - {\tau _x}{N_z}} \right)+\mathbf{k}\left( {{\tau _x}{N_y} - {\tau _y}{N_x}} \right)$$

According to Eqs. (5.1) and (5.3) the Cartesian components of the vectors **N** and **τ** are given by the following formulae

5.5 $${\mathrm{N}_x}=\frac{{ - \partial f/\partial x}}{{\sqrt {1+{{\left( {\partial f/\partial x} \right)}^2}+{{\left( {\partial f/\partial y} \right)}^2}} }}$$

5.6 $${\mathrm{N}_y}=\frac{{ - \partial f/\partial y}}{{\sqrt {1+{{\left( {\partial f/\partial x} \right)}^2}+{{\left( {\partial f/\partial y} \right)}^2}} }}$$

5.7 $${\mathrm{N}_z}=\frac{1}{{\sqrt {1+{{\left( {\partial f/\partial x} \right)}^2}+{{\left( {\partial f/\partial y} \right)}^2}} }}$$

5.8 $${\tau _x}=\frac{{\left( { - \sin \varphi +\frac{1}{R}\frac{{\partial R}}{{\partial \varphi }}\cos \varphi } \right)}}{m}$$

5.9 $${\tau _y}=\frac{{\left( {\cos \varphi +\frac{1}{R}\frac{{\partial R}}{{\partial \varphi }}\sin \varphi } \right)}}{m}$$

5.10 $${\tau _z}=\frac{{\left[ {\frac{{\partial f}}{{\partial x}}\left( {\frac{1}{R}\frac{{\partial R}}{{\partial \varphi }}\cos \varphi - \sin \varphi } \right)+\frac{{\partial f}}{{\partial y}}\left( {\frac{1}{R}\frac{{\partial R}}{{\partial \varphi }}\sin \varphi +\cos \varphi } \right)} \right]}}{m}$$

where

5.11 $$m={\{ 1+\frac{1}{{{R^2}}}{\left( {\frac{{\partial R}}{{\partial \varphi }}} \right)^2}+{\left[ {\frac{{\partial f}}{{\partial x}}\left( {\frac{1}{R}\frac{{\partial R}}{{\partial \varphi }}\cos \varphi - \sin \varphi } \right)+\frac{{\partial f}}{{\partial y}}\left( {\frac{1}{R}\frac{{\partial R}}{{\partial \varphi }}\sin \varphi +\cos \varphi } \right)} \right]^2}\} ^{^{{^{{^{{^{{^{{1/2}}}}}}}}}}}}$$

Similarly to Eq. (4.4), propagation of the flame front in the present case is described by the following kinematic equation 

5.12 $$\frac{{\partial \mathbf{R}}}{{\partial t}}\cdot \mathbf{n}={V_n}$$

Accounting for Eqs. (5.2) and (5.4), the latter equation yields

5.13 $$\begin{gathered} \frac{{\partial \mathrm{R}}}{{\partial t}}\left[ {\left( {{\tau _y}{N_z} - {\tau _z}{N_y}} \right)\cos \varphi +\left( {{\tau _z}{N_x} - {\tau _x}{N_z}} \right)\sin \varphi +\left( {{\tau _x}{N_y} - {\tau _y}{N_x}} \right)\left( {\frac{{\partial f}}{{\partial x}}\cos \varphi +\frac{{\partial f}}{{\partial y}}\sin \varphi } \right)} \right] \hfill \\ \quad \quad ={V_n} \hfill \\ \end{gathered}$$

In the present case, the normal velocity of flame front propagation depends on its curvature as in Eqs. (4.8) and (4.9). It should also depend on the landscape slope, since flame moving uphill is heating the fuel (forest) ahead of it more intensely due to the buoyancy forces than the flame front propagating on a plane. Similarly, the flame moving downhill is heating the fuel (forest) ahead of it less intensely due to the buoyancy forces than the flame front propagating on a plane. According to Ref^45^., the flame propagation up or down the slope changes for the factor $\tt \exp \left[ {sgn\left( {\tan \alpha } \right)3.533{{\left( {\tan \alpha } \right)}^{1.2}}} \right]$ where $\sin \alpha =\mathbf{n}\cdot \mathbf{k}=\left( {{\tau _x}{N_y} - {\tau _y}{N_x}} \right)$. Thus, Eqs. (4.8), (5.4) and (5.13) yield the following dimensionless equation, replacing Eq. (5.13) in the case of a flame front propagating over a hilly landscape

5.14 $$\tt \frac{{\partial \mathrm{R}}}{{\partial t}}=\frac{{\left( {1 - \mu k} \right)\exp \left[ {sgn\left( {\tan \alpha } \right)3.533{{\left( {\tan \alpha } \right)}^{1.2}}} \right]}}{{\left[ {\left( {{\tau _y}{N_z} - {\tau _z}{N_y}} \right)\cos \varphi +\left( {{\tau _z}{N_x} - {\tau _x}{N_z}} \right)\sin \varphi +\left( {{\tau _x}{N_y} - {\tau _y}{N_x}} \right)\left( {\frac{{\partial f}}{{\partial x}}\cos \varphi +\frac{{\partial f}}{{\partial y}}\sin \varphi } \right)} \right]}}$$

It is worth noting that the buoyancy-related empirical factor $\tt \exp \left[ {sgn\left( {\tan \alpha } \right)3.533{{\left( {\tan \alpha } \right)}^{1.2}}} \right]$ included in the normal velocity of flame propagation V_n_ in Eq. (5.14) can include, as an average, the buoyancy-driven flame fingering observed in Ref^74^., which is probably driven by the Rayleigh-Taylor/Rayleigh–Bénard instability inevitable when the hot lighter gas is located below the cold heavier surrounding atmosphere.

The x, y and z coordinates of the flame front denoted as X, Y and Z, respectively, are given by

5.15 $$X=R\left( {t,\varphi } \right)\cos \varphi ,\quad Y=R\left( {t,\varphi } \right)\sin \varphi ,\quad Z=f\left[ {R\left( {t,\varphi } \right)\cos \varphi ,\;R\left( {t,\varphi } \right)\sin \varphi } \right]$$

Accordingly, the curvature of the flame front k is found as^72^

5.16 $${k^2}= \frac{(X'^{2}+ Y'^{2}+ Z'^{2}) ( X''^{2}+ Y''^{2}+ Z''^{2}) - {(X' X'' + Y' Y'' + Z' Z '' )}^2} {{{{( {{X^{'2}}+{Y^{'2}}+{Z^{'2}}} )}^3}}}$$

where primes denote differentiation by φ. Note, that k > 0 when $\partial \boldsymbol{\uptau}/\partial \varphi \cdot \mathbf{n}<0$, and k < 0 when $\partial \boldsymbol{\uptau}/\partial \varphi \cdot \mathbf{n}>0$.

As a particular example of a landscape, consider

5.17 $$z={z_0}\sin \left( {{k_x}x} \right)\sin \left( {{k_y}y} \right)$$

where z_0_ is the length scale, and k_x_ and k_y_ are the wavenumbers of the x and y-directions, respectively.

Render all lengths using z_0_ as the length scale and drop overbars over dimensionless parameters for brevity [in addition, time in Eq. (5.14) was rendered dimensionless by z_0_/V_0_, and µ = L_µ_/z_0_]. Then, Eq. (5.17) yields

5.18 $$X=R\left( {t,\varphi } \right)\cos \varphi ,\quad Y=R\left( {t,\varphi } \right)\sin \varphi$$

5.19 $$Z=\sin \left[ {{K_x}R\left( {t,\varphi } \right)\cos \varphi } \right]\sin \left[ {{K_y}R\left( {t,\varphi } \right)\sin \varphi } \right]$$

5.20 $$\frac{{\partial f}}{{\partial x}}={K_x}\cos \left[ {{K_x}R\left( {t,\varphi } \right)\cos \varphi } \right]\sin \left[ {{K_y}R\left( {t,\varphi } \right)\sin \varphi } \right]$$

5.21 $$\frac{{\partial f}}{{\partial y}}={K_y}\sin \left[ {{K_x}R\left( {t,\varphi } \right)\cos \varphi } \right]\cos \left[ {{K_y}R\left( {t,\varphi } \right)\sin \varphi } \right]$$

with the following dimensionless groups involved

5.22 $${K_x}={z_0}{k_x},\quad {K_y}={z_0}{k_y}$$

The chosen landscape (5.19) is hilly and periodic. It should be emphasized that instead of the landscape (5.19), another landscape, e.g., provided by aerial photography, could be used. Also, the initial condition for Eq. (5.14) can be posed as

5.23 $$t=0,\quad R=\frac{{{R_0}}}{{{z_0}}}\left( {1+\varepsilon \sin n\varphi } \right)$$

Propagation of the flame front over the landscape (5.19) predicted using Eq. (5.14) is illustrated in Fig. 15. It reveals how the sections of the flame front propagating uphill run forward due to the factor $\tt \exp \left[ {sgn\left( {\tan \alpha } \right)3.533{{\left( {\tan \alpha } \right)}^{1.2}}} \right]$ in Eq. (5.14) and develop four distinct tongues of flame.

The propagation of a flame front in a forest fire can also be affected by wind. According to^39^, the normal velocity of a flame front in a forest fire can be expressed as the following function

5.24 $${V_n}={V_{n0}}\left[ {1+a\left( {\frac{{{U_n}}}{{{V_{n0}}}}} \right)+b{{\left( {\frac{{{U_n}}}{{{V_{n0}}}}} \right)}^2}\operatorname{sgn} \left( {{U_n}} \right)} \right]$$

where U_n_ is the projection of the wind velocity at 10 m above the trees onto the normal unit vector to the flame front **n**, and the coefficients a and b are functions of the fuel (forest) type and moisture level. Note that alternative correlations are also available, e.g., in Ref^75^.

The wind velocity vector **U** can be expressed through its components as **U** = U_x_**i**+U_y_**j**. Then, using Eq. (5.4) one finds

5.25 $$\frac{{{U_n}}}{{{V_{n0}}}}=\frac{{{U_x}}}{{{V_{n0}}}}\left( {{\tau _y}{N_z} - {\tau _z}{N_y}} \right)+\frac{{{U_y}}}{{{V_{n0}}}}\left( {{\tau _z}{N_x} - {\tau _x}{N_z}} \right)$$

Accordingly, Eq. (5.14) is now generalized as

5.26 $$\tt \frac{{\partial \mathrm{R}}}{{\partial t}}=\frac{{\left( {1 - \mu k} \right)\exp \left[ {sgn\left( {\tan \alpha } \right)3.533{{\left( {\tan \alpha } \right)}^{1.2}}} \right]\left[ {1+a\left( {{U_n}/{V_{n0}}} \right)+b{{\left( {{U_n}/{V_{n0}}} \right)}^2}\operatorname{sgn} \left( {{U_n}} \right)} \right]}}{{\left[ {\left( {{\tau _y}{N_z} - {\tau _z}{N_y}} \right)\cos \varphi +\left( {{\tau _z}{N_x} - {\tau _x}{N_z}} \right)\sin \varphi +\left( {{\tau _x}{N_y} - {\tau _y}{N_x}} \right)\left( {\frac{{\partial f}}{{\partial x}}\cos \varphi +\frac{{\partial f}}{{\partial y}}\sin \varphi } \right)} \right]}}$$

It is solved with the initial condition given by Eq. (5.23).

Equation (5.26) was semi-discretized on the angular values $\varphi ={\varphi _i}$ (i = 1,…*N* + 1) with φ_1_ = 0 and φ_*N*+1_ = 2π which makes it a system of the inter-related ordinary differential equations (ODEs) in time of the type $d{R_i}/dt=F\left( {{R_{i+1}},{R_{i - 1}},{R_i},{\varphi _i}} \right)$. The inter-related system of (*N* + 1) ODEs is solved numerically starting from the initial condition (5.23) using the Kutta-Merson method.

The results shown in Fig. 15 ascertain an important fact that the configuration of the flame front is practically determined by the presence of the hill slopes where the velocity of flame uphill propagation significantly increases, rather than by the initial flame configuration. In Fig. 15, the flame front, which was initiated in a five-lobe configuration resembling that in Fig. 14a, developed into a four-tongue structure due to the effect of the hills surrounding the ignition area. Accordingly, a flame ignited at the same place but without any lobes (i.e., with n = 0 instead of n = 5) develops practically the same configuration as the flame shown in Fig. 15 and corresponding to n = 5.

**Fig. 16:**
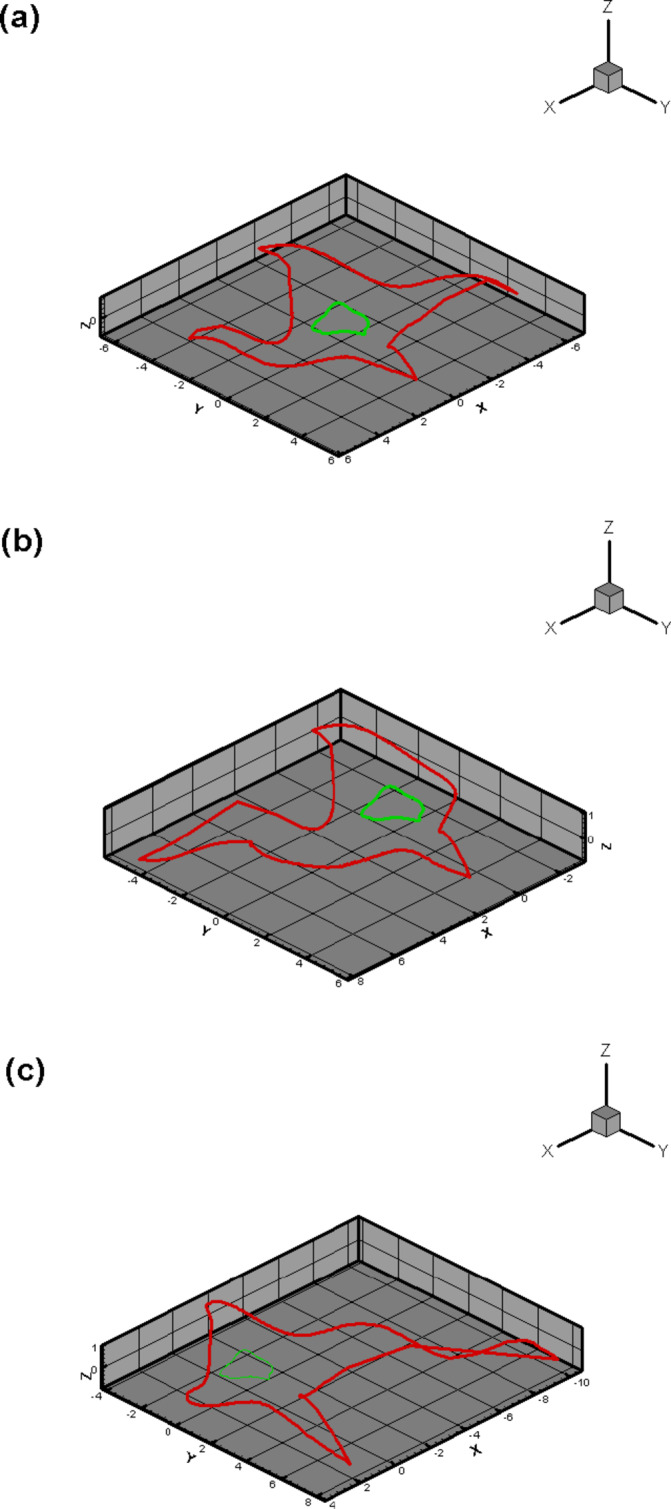

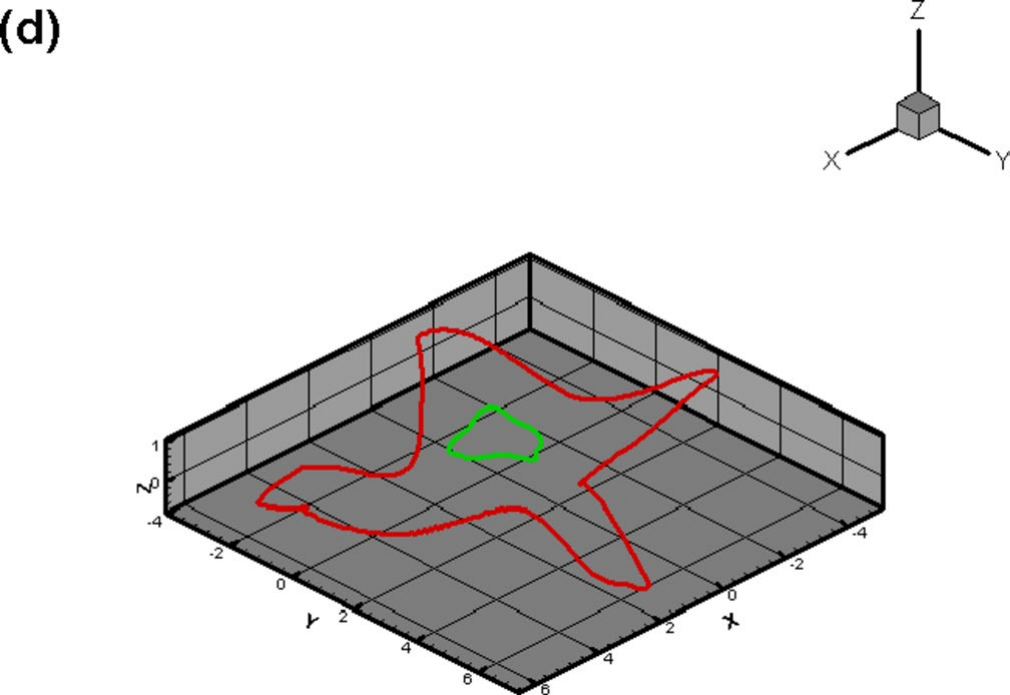
Flame front propagating over the same hilly landscape as the one shown in Fig. 15, from two perspectives (**a**) and (**b**). Green lines show the initial flame front at t = 0 (the spiraling blue lines visualizing the landscape are now omitted so as not to obscure the detailed structure of the flame front). Red lines show the flame front at t = 4. As in Fig. 15, the wavenumber of the initial perturbation *n* = 5, the dimensionless perturbation amplitude ε = 0.1, and $\mu ={10^{ - 3}}$. The dimensionless wavenumbers are K_x_=1 and K_y_=0.5, and the ratio R_0_/z_0_ = 1. (**a**) U_x_/V_n0_=U_y_/V_n0_=0, a = 0.5, b = 0.05; (**b**) U_x_/V_n0_=1, U_y_/V_n0_=0, a = 0.5, b = 0.05; (**c **) U_x_/V_n0_=0, U_y_/V_n0_=1, a = 0.5, b = 0.05; (**d**) U_x_/V_n0_=1, U_y_/V_n0_=1, a = 0.5, b = 0.05.

The predicted effect of wind on the flame propagation and configuration is illustrated in Fig. 16. In particular, the flame configuration in Fig. 16a repeats the result of Fig. 15 when the wind velocity is zero, i.e., U_x_=U_y_=0. In Fig. 16b, wind is blowing in the direction of the x-axis, i.e., U_x_/V_n0_=1, U_y_/V_n0_=0, while in Fig. 16c, wind is blowing in the direction of the y-axis, i.e., U_x_/V_n0_=0, U_y_/V_n0_=1. In addition, Fig. 16d shows the flame front in the case of the wind blowing by diagonal between the x- and y-axes, with U_x_/V_n0_=1, U_y_/V_n0_=1. It is seen that the interaction of the effects of the wind and the hilly landscape results in complicated configurations of forest fire fronts, when one of the flame tongues can suddenly extend significantly in a certain direction.

**Fig. 17:**
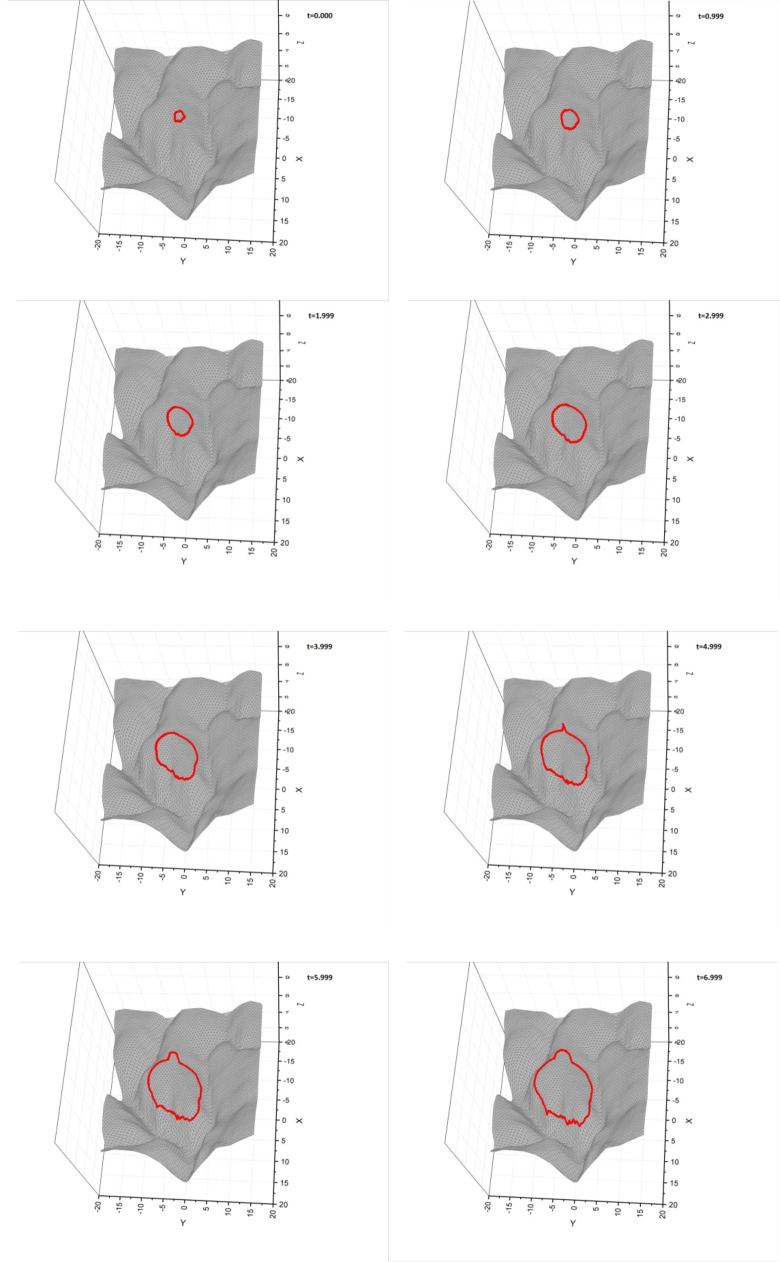

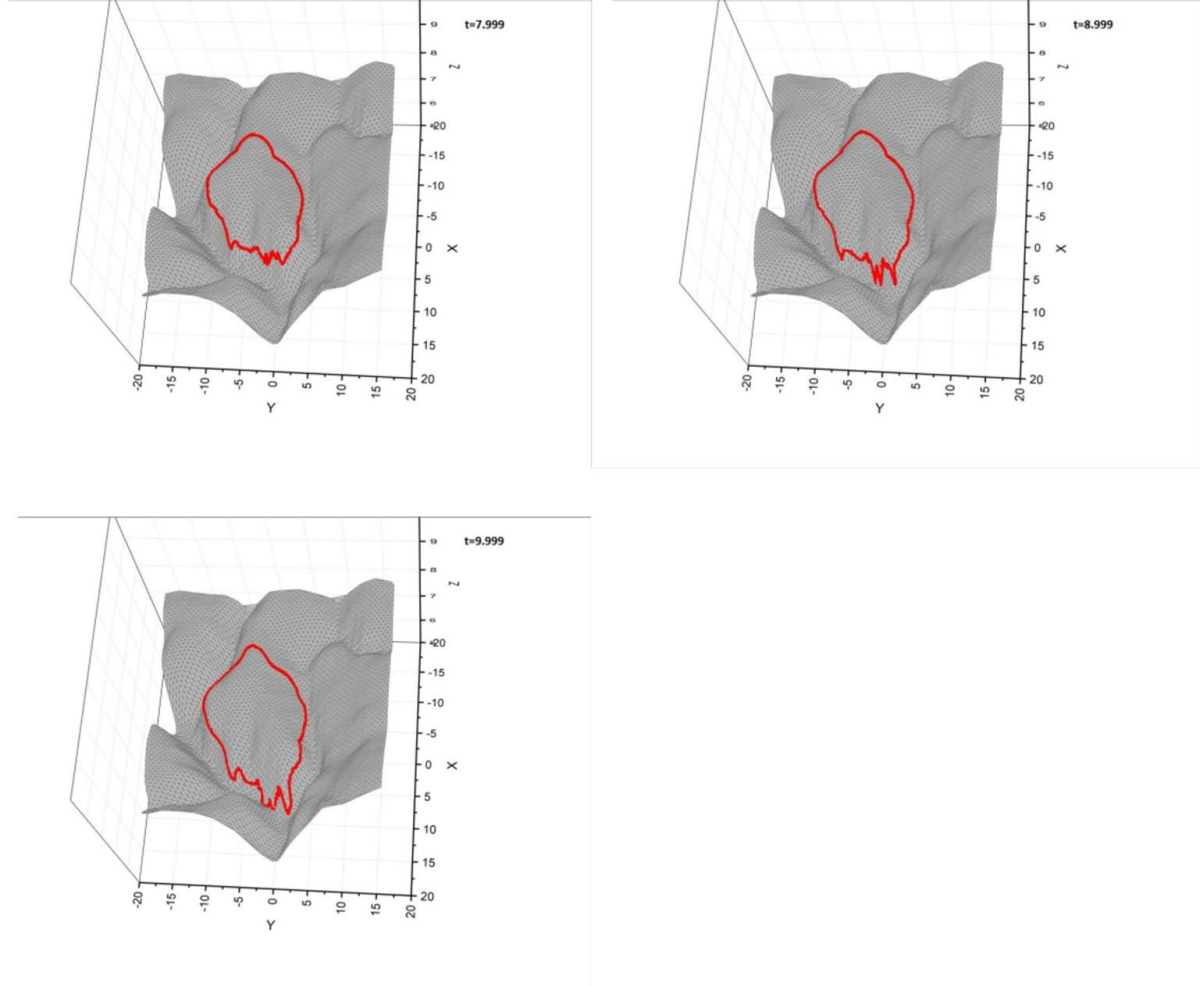
Predicted flame front propagating over a real landscape without wind. Consecutive dimensionless time moments are listed in the top-right corners of the panels.

Several snapshots from the forest flame propagation event predicted by the present method on a real landscape under realistic conditions are presented in Fig. 17.

Finally, note that the characteristic size of the real landscape presented in Fig. 17 is kilometers. Still, at the present stage when the data on forest fires frequently do not include either the type of burning vegetation (the volatile and water content in the wood), or a detailed evolution of the flame front in time, or the wind pattern, etc., it is preferable to explore the present model in dimensionless form, as in Fig. 17, not attempting yet to fit the scattered and incomplete empirical information. The same approach to the interpretation of the predictions of the evolution of planar flame fronts was taken, for example, in Ref^73^.

## Conclusion

In the present work, the flame propagation was studied experimentally and the flame propagation velocities affected by the spatial step between the flame sources, the inclination angles and the existence of wind were calculated. Four spatial step experiments were conducted with 9 mm, 11 mm, 13 mm, 15 mm, and the flame propagated at six different inclination angles: 0^ο^, 13.5^ο^, 22.3^ο^, 31.0^ο^, 40.4^ο,^ and 53.9^ο^. The flame source utilized was either a pure cotton swab or a cotton swab dipped in ethanol. The flame propagated with nearly constant velocity (cf. Fig. 4). The transportation morphologies were recorded by the DSLR camera and FLIR camera and presented as image sequences in Figs. 2, 3, 7, 9, 11 and 12. The obtained velocity values under various conditions are displayed in Figs. 4, 5, 6, 8 and 10, and 13.

In addition, a novel quasi-physical approach for predicting forest flame front propagation over a hilly landscape under the effect of wind is developed, and some characteristic cases are illustrated in Figs. 14, 15 and 16. The predicted results for the flame propagation over a real landscape without wind are presented in Fig. 17.

The key findings reveal the dramatic effect of wind on flame propagation velocity and the front configuration, and the fundamental difference between highly flammable and less flammable fuels. One of the main theoretical conclusions is that the front configuration of the flame is dominated by topography and wind more than by the initial ignition configuration.

The broader physical interpretation of the present experimental and theoretical results is intrinsically linked to the application of the dimensional analysis. The present work developed a quasi-physical model where all the phenomena determining the flame velocity without the effects of buoyancy and wind are lumped into the two model parameters: V_n0_ and µ. As stated below Eq. (4.11), the Markstein correction µ is already a dimensionless parameter, µ = L_µ_/R_0_, where L_µ_ is the Markstein length, and R_0_ is the characteristic radius of curvature scale associated with the original flame front. The dependence of the flame velocity V_n0_ on the relevant dimensionless parameters governing flame spread can be found in Ref.^76^. Additionally, the flame velocity V_n0_ is dependent on volatile and water content in a particular wood, and the dimensionless parameters which stem from the theory of their eruptions in flame in Ref.^9^. The buoyancy factor $\tt \exp \left[ {sgn\left( {\tan \alpha } \right)3.533{{\left( {\tan \alpha } \right)}^{1.2}}} \right]$ used here was provided in Ref.^45^ for a particular case, whereas its generalization should inevitably involve the Froude number, the Rayleigh number and the Grashof number^75,76^. The wind-related factor in Eq. (5.24) should be determined by the Reynolds number, the dimensionless terrain curvature scale, and several other dimensionless groups associated with the non-isothermal turbulent gas flows^76^.

## Perspectives and future work

It should be emphasized that the inception of the flame front of forest fire was explored in model experiment and the theory in the recent work of the present group^57^. The model of propagation of continuous flame front developed in the present work is a good approximation of a situation where the distance between trees in forest fire and the characteristic fire transfer time from tree to tree are significantly smaller and shorter, respectively, than the characteristic size of the forest fire or its characteristic evolution time. The model developed in the present work aims at large-scale forest fires rather than the model qualitative experiments here. Fitting the model parameters such as V_n0_ or µ is currently hardly possible because the available published data on forest fires typically lack many important details in their totality, like slopes and wind speed, the type of wood involved, the volatile and water content in the wood and so on. Accordingly, future work would aim at an extension of the approach of the model experiments to real-scale observations of natural forest fires. That would require processing data from many experimentally documented and some numerically predicted cases of forest fire propagation to extract the lump parameters involved in this simplified quasi-physical model in totality. Such massive data analysis would probably involve application of AI. Establishment of the lump parameters involved in the model will allow its application in real time when an ignition source is detected, the known local topography is fed in the model code, as well as the current predominant wind direction and speed. Then, firefighters will be able to predict the forthcoming directions and configurations of the flame front and the overall pattern of its propagation using a laptop in real time and take appropriate preventive measures. Moreover, multiple scenarios of local forest fire developments can be predicted beforehand, and thus, preventive forestry and safety measures could be taken.

## Data Availability

Data is available from the authors upon reasonable request.
